# Synergistic enhancement of efficacy of platinum drugs with verteporfin in ovarian cancer cells

**DOI:** 10.1186/s12885-020-06752-1

**Published:** 2020-04-03

**Authors:** Venkata Ramesh Dasari, David J. Carey, Radhika Gogoi

**Affiliations:** 1 Department of Molecular and Functional Genomics, Weis Center for Research, Geisinger Medical Center, Danville, PA USA; 2 Department of Women’s Health, Geisinger Medical Center, Danville, PA USA

**Keywords:** Ovarian cancer, Verteporfin, Cisplatin, Carboplatin, Paclitaxel, Cell cycle, Cytokines

## Abstract

**Background:**

Epithelial ovarian cancers (EOCs) comprises the majority of malignant ovarian neoplasms. Combination treatment with chemotherapeutic agents seems to be a promising strategy in ovarian cancer (OVCA) patients in order to overcome drug resistance. In this in vitro study, we investigated the therapeutic efficacy of verteporfin (VP) alone and in combination with cisplatin (CDDP), carboplatin (CP) and paclitaxel (Taxol). The main objectives of this study are to determine the nature of interactions between VP and CDDP/CP/Taxol and to understand the mechanism of action of VP in OVCA cells.

**Methods:**

The efficacy of VP on cell proliferation, cytotoxicity, invasion and clonogenic capacity was assayed in CDDP-sensitive (COV504, OV-90) and CDDP-resistant (A2780Cis) cell lines. The cytotoxic effects of drugs either alone or in combination were evaluated using MTT assay and Cell Viability Blue assay. The effects of drugs on the metabolic functions were studied using matrigel invasion assay and clonogenic assay. Immunoblot analysis was carried out to investigate changes in YAP and cell cycle genes. Changes in the cytokines due to drug treatments were analyzed using a cytokine array.

**Results:**

Treatment with VP inhibited cell proliferation, invasion and increased cytotoxicity of OVCA cells. We observed that VP chemosensitized CDDP-resistant cells, even at lower doses. When added either in constant or non-constant ratios, VP produced synergistic effects in combination with CDDP/CP/Taxol. A cytokine array identified upregulation of cytokines in OVCA cells that were inhibited by VP treatment.

**Conclusions:**

Either in cisplatin-resistant cell lines or cisplatin-sensitive cell lines, VP proves to be more efficient in inhibiting cell proliferation and inducing cytotoxicity. Our results suggest that novel combinations of VP with CDDP or CP or Taxol might be an attractive therapeutic strategy to enhance OVCA chemosensitivity. The fact that lower doses of VP are effective in chemosensitizing the CDDP-resistant cells, might ultimately lead to the development of an innovative combination therapy for the treatment of OVCA patients.

## Background

Epithelial ovarian cancers (EOCs) comprises the majority (about 95%) of malignant ovarian neoplasms [1–4]. EOC has been conventionally treated with cytoreductive surgery followed by platinum- and taxane-based chemotherapy. Even with advanced techniques in surgical debulking, optimization of chemotherapeutic regimens, and improvements in radiotherapy, 5-year progression free survival (PFS) and overall survival (OS) rates remain low [5–7]. Most women with high-grade serous ovarian cancer (HGSOC) initially respond well to chemotherapy treatment; however, most develop chemoresistance [8, 9]. Chemotherapeutic resistance has been a challenge in the treatment of OVCA, especially the HGSOC. Recurrent tumors are characteristically more resistant to chemotherapy, with lower response rates [10, 11]. The limited efficacy of chemotherapy in recurrent disease has been attributed to the development of multiple-drug resistance (MDR) [11–15]. Studies by Ozols et al., [16] demonstrated that carboplatin is as effective as cisplatin and is better tolerated. The current consensus standard for chemotherapy is a combination of carboplatin and paclitaxel, both administered every 3 weeks, or carboplatin every 3 weeks and paclitaxel weekly, in a dose-dense manner. The regimen is generally well tolerated but is associated with several side effects [4]. Hence, improving OS of EOC patients depends on augmenting chemotherapeutic strategies to overcome the drug resistance as well as to regulate the development of drug resistance. The primary objective of this investigation is to identify new chemotherapeutic agents for the treatment of OVCA in order to regulate platinum drug-resistance in OVCA cells.

Yes-associated protein (YAP) is a potent transcription coactivator acting via binding to the TEAD transcription factor and plays a critical role in organ size regulation. YAP is phosphorylated and inhibited by the Lats kinase, a key component of the Hippo tumor suppressor pathway [17]. Verteporfin (VP) [18], an FDA approved drug used in photodynamic therapy (PDT) for adult macular degeneration was recently identified as an inhibitor of YAP and its binding to its partner TEA Domain Transcription Factor 1 (TEAD) [19]. Since the identification of VP as a YAP/TEAD inhibitor, several in vitro and in vivo studies have revealed the potential of VP for treatment of different cancers [20–23]. We tested the efficacy of VP treatment in Type 1 endometrial cancer (EMCA) cells (HEC-1-A and HEC-1-B) and observed cytotoxic and anti-proliferative effects [24] and analyzed RNAseq data to investigate the comprehensive transcriptomic landscape of VP treated Type 1 EMCA cells [25]. We also observed that subcutaneous tumors of EMCA in nude mice were regressed after VP treatment by inhibiting cell cycle pathway proteins. Extrapolating our previous results with EMCA, in this study we report the efficacy and synergistic activity of VP with other chemotherapeutic drugs cisplatin (CDDP), carboplatin (CP) and paclitaxel (Taxol) in serous ovarian cancer (OVCA) cells.

## Methods

### OVCA cell lines and culture conditions

We used two platinum-sensitive cell lines, OV-90 and COV504 and one platinum-resistant cell line (A2780Cis). OV-90 cells were grown in 1:1 mixture of MCDB105 medium and Medium 199, supplemented with 15% (v/v) fetal bovine serum (FBS). COV504 cells were grown in DMEM (1X) medium supplemented with 10% (v/v) FBS. A2780Cis cells were grown in RPMI medium supplemented with 10% (v/v) FBS. All culture media were supplemented with 1% Antibiotic-Antimycotic and were incubated at 37 °C in a humidified atmosphere containing 5% carbon dioxide. All cell lines used in the study were between 15 and 25 passages. Details of the cell lines were given in Supplementary Table S1a and details of the media, FBS and antibiotic-antimycotic were given in Supplementary Table S1b.

### Drug treatments

Verteporfin and Paclitaxel were dissolved in DMSO and added to the medium. Cisplatin was dissolved in sterile PBS and added to the medium. Carboplatin was dissolved in sterile water and added to the medium. Controls cells were treated with equal concentrations of vehicles (DMSO or sterile PBS or sterile water). Sources of drugs were detailed in Supplementary Table S2. The IC_50_ (50% inhibitory concentration) values were calculated based on Chou-Talalay method [26] using Compusyn software.

### MTT assay and calculation of IC_50_ values

The effect of drugs on cell proliferation was determined by using MTT assay kit (Sigma, Supplementary Table S3) as per manufacturer instructions. Briefly, cells were plated at 5000/well or 10,000/well in 200 μl of complete culture medium containing different concentrations of drugs (as described in Results) in 96-well microtiter plates for 72 h at 37 °C in a humidified chamber. After 72 h, 10 μl of MTT labeling reagent was added to each well, and the microplates were incubated for 4 h in humidified atmosphere (37 °C, 5% CO_2_). Then 100 μl of the solubilization solution was added to each well and incubated overnight in humidified atmosphere. Absorbance was recorded on a microplate reader at 550 nm wavelength with reference wavelength at  690 nm. The effect of the drugs on proliferation was assessed as the percentage of inhibition in regard to the untreated controls (100%). For each drug, we constructed a standard curve using MTT assay, and these were used to calculate IC_50_ values using Compusyn software following Chou-Talalay method [26].

### Cell viability assay

The CellTiter-Blue® Assay (Promega) is based on the ability of living cells to convert a redox dye (resazurin) into a fluorescent end product (resorufin). OVCA cells were plated in 96-well microtiter plates at a final concentration of 5000 or 10,000 cells/well. Following treatment with drugs for different periods, CellTiter-Blue reagent was added, and the plates were incubated at 37 °C for 1–2 h for color development and fluorescence read at 560/590 nm. Cytotoxicity values of the drugs were calculated from cell viability values.

### Synergy determination among drugs

The Isobologram analysis for the combination study was based upon the Chou-Talalay method to determine combination indices (CI). The data obtained with the MTT assay was normalized to the vehicle control. Then, the data was converted to Fraction affected (Fa; range 0–1; where Fa = 0 represents 100% viability and Fa = 1 represents 0% viability) and analyzed with the CompuSyn™ software (http://www.combosyn.com/) based upon the Chou and Talalay median effect principle [27, 28]. The CI values reflect the ways of interaction between two drugs. CI < 1 indicates synergism, CI = 1 indicates an additive effect, and CI > 1 indicates antagonism. Dose-Reduction Index (DRI) is defined as a measure of how many folds the dose of each drug in a synergistic combination may be reduced at a given effect level when compared with the doses of each drug alone. The concentrations of the drugs used in the study were described in Results.

### Western blot analysis

Cells were treated with either drugs or vehicles for various time periods as described in the Results. After the treatment period, cells were lysed in RIPA buffer supplemented with protease and phosphatase inhibitors and subjected to SDS-PAGE. Samples were separated electrophoretically on 10 to 12% gels, electroblotted onto nitrocellulose membrane (Bio-Rad), blots were blocked at room temperature for 1 h in 5% (w/v) milk in phosphate-buffered saline and incubated overnight at 4 °C with primary antibodies. Details of primary and secondary antibodies used in the study are provided in Supplementary tables S4a and S4b. Protein bands were visualized with an enhanced chemiluminescence substrate (Pierce Biotechnology) and detected using LAS-3000 (Fujifilm, Tokyo, Japan). Full-length blots are presented in Supplementary Figure S9.

### Invasion assay

Transwell invasion assays were carried out using 8.0 μm cell culture inserts in 24-well plates. The upper surface of filters was precoated with extracellular matrix coating (Matrigel). After treatment with either DMSO control or drug at Fa0.5 (see Supplementary Table S6), cells were washed twice with sterile 1x PBS to remove the dead cells, harvested and counted using Cellometer AutoT4 (Nexcelom Bioscience) counter. 100,000 viable cells in serum-free medium were seeded on to the upper chamber of each insert, with complete medium added to the bottom chamber. Following incubation, invasive cells on the lower surface of the filters were fixed and stained with the Differential Quik Stain Kit (Electron Microscopy Sciences) and counted.

### Clonogenic assay

Survival following drug exposure was defined as the ability of the cells to maintain their clonogenic capacity. Briefly, increasing numbers of cells (200, 400, 800) treated with drugs (Fa0.1) (see Supplementary Table S6) for 24 h were plated in 6-well plates. Colonies formed were fixed and stained with a solution containing 4% formaldehyde and 1% crystal violet and those with at least 50 cells were counted by two independent blinded investigators. The number of colonies obtained from three replicates was averaged for each condition. These mean values were corrected according to plating efficiency of respective controls to calculate cell survival for each dose level. The linear quadratic equation was fitted to data sets to generate survival curves, and dose enhancement factor was calculated at 10% surviving fraction (DEF 0.1) [29].

### Cytokine array analysis

Cytokine levels in control and VP-treated samples were determined using human cytokine antibody array (Ray Biotech, Cat. No. AAH-CYT-5-8) (Supplementary Table S3) as per manufacturer instructions. Using this array, we assayed the expression of 80 cytokines in OVCA cell lines. Briefly, the membranes from the cytokine array kit were incubated with control and VP (Fa0.5) treated cell lysates (500 μg of total protein) overnight at 4 °C (n=1). The membranes were then processed as per manufacturer and then assayed using chemiluminescence technique. Spots were identified and local background subtracted. By comparing the signal intensities, relative levels of cytokines were established.

### Statistical analysis

All experiments were repeated at least 3 times (with triplicates) unless otherwise noted. Data are presented as Mean ± SEM unless otherwise noted. Data were analyzed for significance using one-way analysis of variance (ANOVA) using Graph Pad Prism software or MS Excel Office 365. Results were considered statistically significant at a *p* < 0.05 (Vehicle treated vs drug treated).

## Results

### Verteporfin -single and combination studies on OVCA cells

The main objective of the present study is to investigate the effect of VP on OVCA cell lines, with special focus on VP-platinum (CDDP, CP) and VP-Taxol drug combinations. We used two types of cell lines: CDDP-sensitive (OV-90, COV504) and CDDP-resistant (A2780Cis) cell lines. Cells were exposed to different concentrations of VP, CDDP, CP and Taxol for different time periods and standard curves and dose-effect curves were constructed using MTT analysis following the Chou-Talalay method [26] (Supplementary Figs. S1, S2). As expected, CDDP had higher IC_50_ values in A2780Cis compared to CDDP-sensitive cell lines (Table 1) consistent with the platinum resistant nature of the cell line. Comparatively, CP showed higher IC_50_ value in COV504 cells and VP and Taxol showed higher IC_50_ values in OV-90 cells. An important point of this study is that CDDP-resistant cell line A2780cis recorded the lowest IC_50_ value for VP compared to other two cell lines. Previously, we reported the effect of VP on two endometrial adenocarcinoma (EMCA) cell lines (HEC-1-A and HEC-1-B) and patient derived organoids [24]. Based on this premise, we also calculated the IC_50_ values of the above drugs in serous EMCA cell lines ARK1 and ARK2 (Supplementary Figs. S3, S4 and Supplementary Table S5). Since the drugs in the present study act via distinct mechanisms, we sought to understand the nature of the interaction between VP and CDDP/CP/Taxol based on the combination index (CI) values [26]. The CI results are shown as heat maps where the green color indicates synergism (CI value < 1), the yellow color indicates additive effect (CI = 1) and the red color indicates antagonism (CI > 1). The drugs were added in constant-ratio and the effects were studied after 72 h of treatment.

**Table 1:** IC_50_ values (in μM) of OVCA cell lines

Cell lines → Drug↓	Cisplatin-sensitive		Cisplatin-resistant
	OV-90	COV504	A2780Cis
Carboplatin (CP)	96.208	117.231	95.53
Cisplatin (CDDP)	2.16766	1.79559	5.1469
Paclitaxel (Taxol)	89.0464	7.164	81.9158
Verteporfin (VP)	29.3327	8.37177	3.83663

**Fig. 1:**
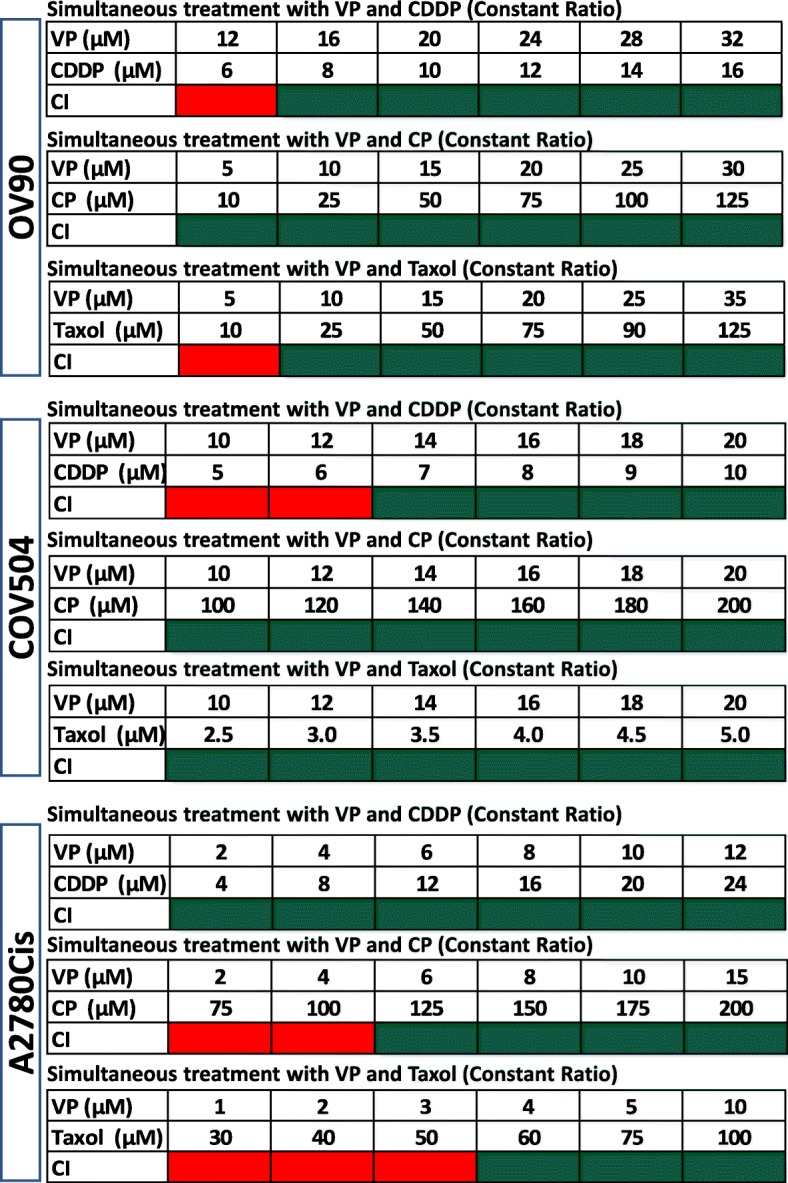
Synergistic activity of drugs on OVCA cell lines: IC_50_ values were calculated using Compusyn software following Chou-Talalay method. These calculations were based on MTT assay which was done in 96-well plates. In each well 5000 cells were seeded. The next day, VP and CDDP/CP/Taxol treatments were initiated and given for 72 h and cell proliferation was measured as per Manufacturer’s instructions (Cell Proliferation Kit). DMSO/sterile PBS /sterile water served as control. *n* = 6. VP = Verteporfin; CDDP = cisplatin; CP = carboplatin; Taxol = paclitaxel. After determining cell proliferation (MTT assay) of OVCA cells treated with constant ratios of VP and CDDP/CP/Taxol, combination index (CI) values were calculated and represented as heat maps (Microsoft Excel Office 365) where a drug combination is synergistic (green color) if CI < 1.0; additive (yellow color) if CI = 1.0; and antagonistic (red color) if CI > 1.0

In VP-CDDP combinations, lower drug combinations are antagonistic, whereas higher drug combinations are synergistic in OV-90 and COV504 cells (Fig. 1, Supplementary Fig. S5). Surprisingly, in CDDP-resistant cell line (A2780Cis), even lower dose-combinations of VP and CDDP found to be synergistic compared to other two cell lines. In VP-CP combinations, lower drug combinations of VP and CP were synergistic in OV-90 cells. Comparatively, in COV504 and A2780Cis cells, higher drug-combinations were synergistic. When compared to CDDP (4 to 24 μM), we used higher concentrations of CP (75 to 200 μM) for combination treatments with VP to produce synergistic effects. These results suggest that CDDP is more effective than CP in inhibiting cell proliferation. In VP-Taxol combinations, we used lowest doses of Taxol to produce synergistic effects in combination with VP in COV504 cells. In case of OV90 and A2780Cis cells, lower drug-combinations were antagonistic and higher drug-combinations were synergistic in inhibiting proliferation of cells. Similarly, we also observed that VP is showing synergistic activity with CDDP, CP and Taxol in EMCA cell line ARK1 (Supplementary Figure S5). The constant ratio of drug combinations provides the most useful information while minimizing the number of drug combination data points. It is the most efficient and cost-effective method, particularly important for in vivo or clinical studies. In designing experiments with non-constant drug ratio, data in each series carry different levels of synergistic effects [26]. Since, we studied the effect of drug combinations using constant ratios, we also studied the effect of these drugs in non-constant combinations in COV504 cells (Supplementary Fig. S6). In these cells, lower doses of VP-CDDP and VP-CP combinations were antagonistic and higher dose combinations were synergistic. We also used higher doses of CP (up to 350 μM) compared to CDDP (up to 25 μM) in combination with VP. On the other hand, lower dose drug combinations of VP-Taxol were found to be synergistic and higher dose combinations were antagonistic in inhibiting cell proliferation. In summary, our study shows that synergy was observed in different doses of drugs with VP irrespective of whether they are CDDP-sensitive or CDDP-resistant cells.

**Fig. 2:**
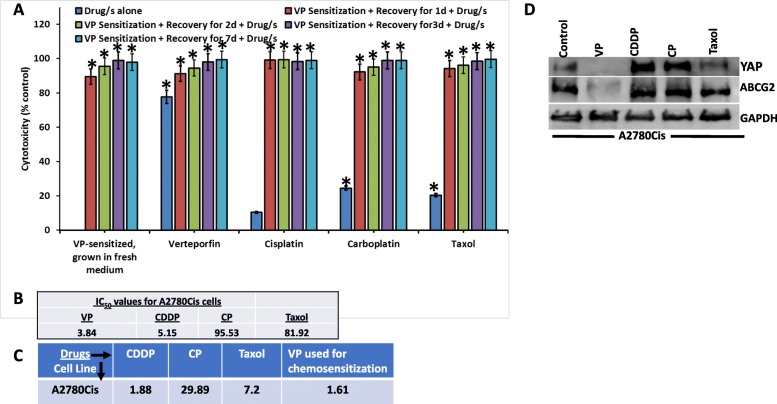
Effect of VP sensitization on cytotoxicity of A2780Cis cells: **a**. Bar graph showing cytotoxicity after chemosensitization. Cells were counted and seeded in 96-well plates (5000cells/well). Treatment conditions: *Drugs alone*: Cells were grown in fresh medium for 24 h and then drugs were added. *VP Sensitization*: Cells were treated with VP (Fa0.1) for 6 h. After 6 h, cells were washed with fresh medium 3X, lifted, counted and seeded in 96-well plates (5000cells/well). Cells were grown in fresh media for 1d/2d/3d/7d and then drugs were added at Fa0.1. Drug treatments are given for 24 h. Cytotoxicity values were based on cell viability assays (CellTiter-Blue®). Bars represent Mean ± SEM. n = 6. * Significant at *p* < 0.05 (1-way ANOVA, Control vs drug dose). VP = Verteporfin; CDDP = Cisplatin; CP = Carboplatin; Taxol = Paclitaxel. **b**. Actual IC_50_ values of A2780Cis cells. **c**. Concentrations of drugs used for the assay. **d**. Western blots showing the effect of drugs on YAP and ABCG2 in A2780Cis cells. Equal amounts of proteins (40 μg) from untreated and treated A2780Cis cell lysates were loaded on 10% gels and transferred onto nitrocellulose membranes, which were then probed with respective antibodies. GAPDH was used a positive loading control. *n* = 3

**Chemosensitization of CDDP-resistant (A2780Cis) cells**: We next asked, whether low dose VP could sensitize A2780Cis, a platinum resistant cell line to platinum therapy. A2780Cis cells were treated with low dose VP (Fa0.1) for 6 h followed by recovery for 1, 2, 3, or 7 days followed by treatment with CP or CDDP or Taxol for 24 h. The concentrations of the drugs used were documented in supplementary table 6. To remove residual effects of VP after treatment for 6 h, the cells were washed 3 times with medium, removed, counted and re-plated before platinum or Taxol drug treatment. These results were compared to those obtained without VP sensitization (drugs alone). Our data demonstrates resensitization to chemo treatment as demonstrated by an increase in cytotoxicity when the A2780Cis cells were sensitized with VP prior to treatment with CP/CDDP/Taxol (Fig. 2). This was observed even after 7d of recovery following VP treatment. We also analyzed the effect of these drugs on the expression of YAP and the drug resistance marker ABCG2 in CDDP-resistant A2780Cis cells. ABCG2 was highly expressed in the control A2780Cis cells (as these cells are CDDP-resistant). However, ABCG2 was inhibited by VP, consistent with inhibition of YAP activity by VP (Fig. 2d). These results suggest that VP sensitizes the cells for effective chemotherapeutic treatment. Based on these results, we hypothesized that lower doses of VP could be effective in chemosensitizing CDDP-resistant cells at calculated Fa0.1 values.

### Efficiency of VP in inhibiting metabolic functions of OVCA cells

**Fig. 3:**
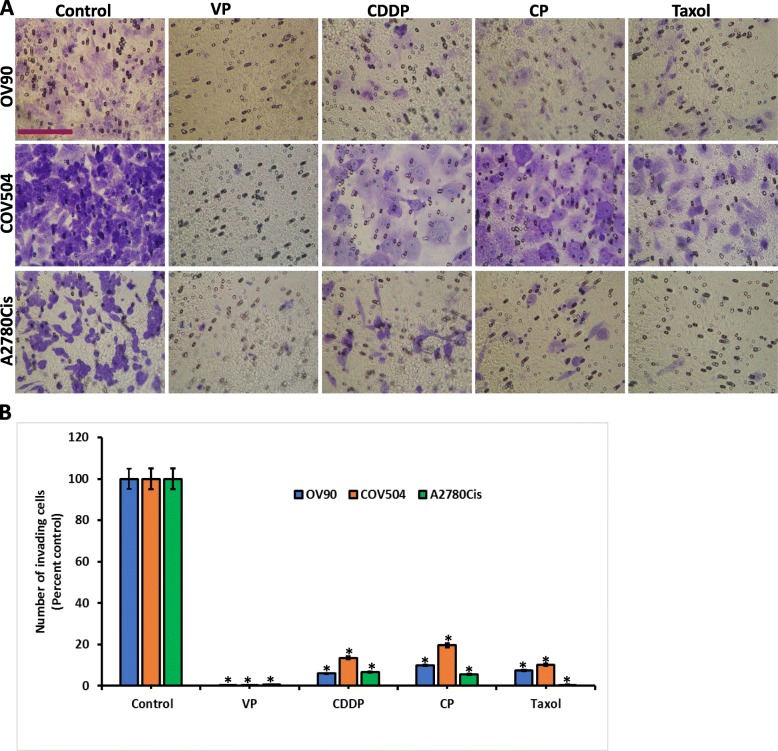
Inhibition of invasion of OVCA cells by drugs. **a**. OVCA cell lines (each 100,000) were treated with vehicle, VP/CDDP/CP/Taxol at Fa0.5 for 72 h. After treatment, cells were lifted, counted and equal number of cells were allowed to invade through the Matrigel for 36 h. Transwell cell inserts with 8 μm pores were used. *n* = 9. Bar = 200 μM. **b**. Quantitative estimation of matrigel invasion assay. Error bars indicate Mean ± SEM. *Statistically significant at p < 0.05 (ANOVA), control vs drug treatment. Data in each group were compared to control cells treated with vehicle. Experiment is repeated 3 times with at least 3 replicates for each cell line

**Fig. 4:**
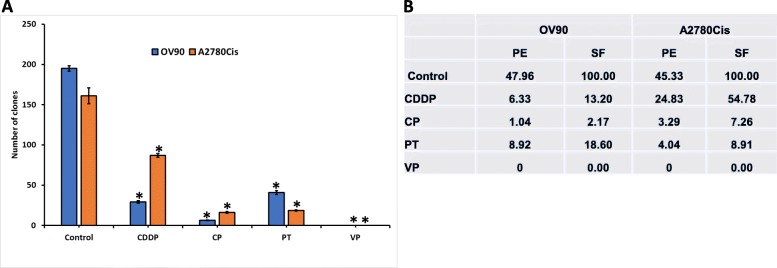
Inhibition of clonal formation after drug treatments: **a**. Bar graph showing the number of clones formed in control and drug treatments. Error bars indicate Mean ± SEM. *Statistically significant at *p* < 0.05 (ANOVA), control vs drug treatment. Data in each group was compared to control cells treated with vehicle. Experiment is repeated 2 times with at least 3 replicates for each cell line. **b**. Table showing the plating efficiency (PE) and surviving fractions (SF) of the cell lines in the present study

We next investigated the effect of VP on OVCA cell invasion. To ensure that equal numbers of viable cells were plated in the drug treated and control groups, cells were treated with drugs (Fa0.5) (see Supplementary Table S6) for 24 h, counted and an equal number of viable cells were plated on Boyden chambers coated with matrigel. Our results demonstrated a significant decrease in invasion in the VP treated group compared to other drugs and untreated cells (Fig. 3a). These results suggest that VP is effective at Fa0.5 values compared to other drugs. (Fig. 3b). Further, we tested the clonogenic capacity of OVCA cells after treatments with drugs at Fa0.1 concentration. Either in OV90 or A2780Cis, CP was more effective in inhibiting clonal capacity than CDDP. Taxol was more effective in A2780Cis cells than OV90 cells. However, VP was more effective in inhibiting clonal expansion of OVCA cells (Fig. 4a, Supplementary Fig. S7). This is reflected in plating efficiency (PE) and surviving fraction (SF) of OVCA cells (Fig. 4b). These results show that VP is more effective than other drugs of study in inhibiting either invasion or clonal capacity of OVCA.

**Fig. 5:**
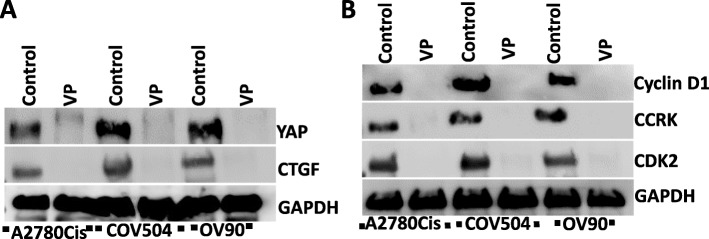
Effect of drugs on metabolic pathway proteins of OVCA cells: Western blots showing the effect of drugs on pathway proteins of OVCA cells. Equal amounts of proteins (40 μg) from untreated and treated OVCA cell lysates were loaded on 10 to 12% gels and transferred onto nitrocellulose membranes, which were then probed with respective antibodies. The westerns were run on separate blots. They were reprobed with GAPDH which was used a positive loading control. *n* = 3. **a**. Effect of VP on YAP and CTGF of OVCA cells. **b**. Effect of VP on cell cycle proteins of OVCA cells

Since we observed inhibition of invasion and clonal capacity by VP, we next investigated the effects of VP on YAP activity in OVCA cells. YAP is part of the HIPPO pathway that induces expression of CTGF. Western analysis of cell lysates of OVCA showed that YAP and CTGF are inhibited by VP treatments (Fig. 5a). Previously, we showed that VP inhibits expression of cell cycle genes in vitro and in vivo in EMCA [24, 25]. Similar to these results, VP inhibits cell cycle gene expression in OVCA also (Fig. 5b).

**Fig. 6:**
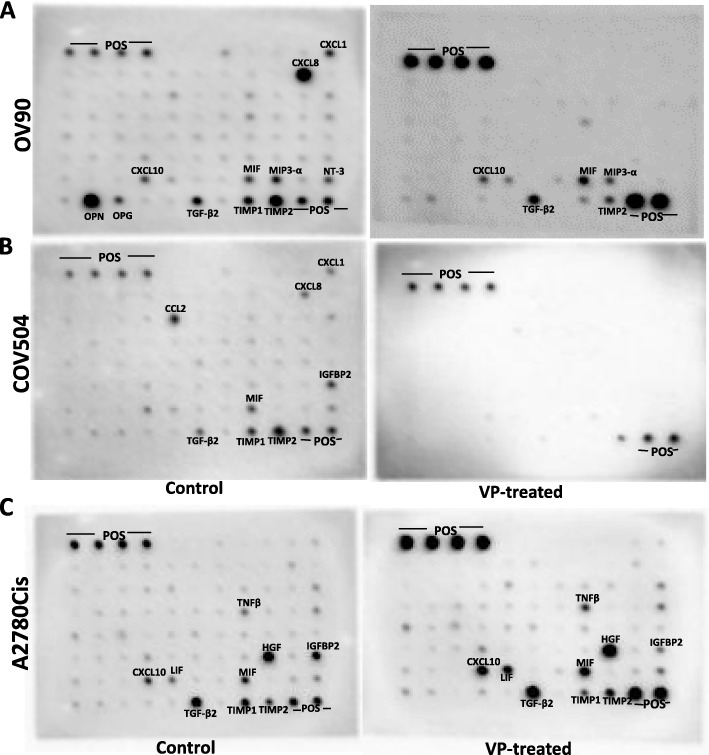
Analysis of cytokines in VP-treated cells: Cytokine levels in control and VP-treated samples were determined using human cytokine antibody array as per manufacturer instructions. Using this array, we performed the proteomic analysis of cytokines and screened the expression of 80 cytokines in OVCA cell lines. The membranes were incubated with cell lysates, then processed and assayed using chemiluminescence technique. Spots were identified and based on the signal intensities; relative levels of cytokines were established

Finally, cytokine levels in control and VP-treated samples were determined using a human cytokine antibody array that assays the expression of 80 cytokines. In OV90, expression of Gro alpha (CXCL1), IGFBP2, IL-8 (CXCL8), IP-10 (CXCL10), LIF, MCP1, MIF, MIP3-α, NT-3, OPG, OPN, TGFβ2, TIMP1, TIMP2, and TNFβ, was inhibited by VP (Fig. 6a). In COV504, expression of Gro alpha, HGF, IGFBP2, IL-8, IP-10, LIF, MCP1, MIF, MIP3-α, NT-3, OPG, OPN, TGFβ2, TIMP1, and TNFβ was inhibited by VP treatment (Fig. 6b). Cytokine HGF was not significantly expressed in OV90 cells and TIMP2 was not expressed in COV-504 cells. In contrast, in A2780Cis cells, expression of cytokines Gro alpha, IL-8, MCP1, MIP3-α, NT-3, OPG, OPN, TIMP1 and TIMP2 was inhibited by VP treatment. Interestingly, we observed the increased expression of cytokines HGF, IP-10, LIF, MIF, TGFβ2 and TNFβ after VP treatment. Compared to other two CDDP sensitive cell lines, there was no significant expression of IGFBP2 in the CDDP-resistant cell line (Fig. 6c). These results show differential expression of cytokines and their response to VP in CDDP-sensitive and CDDP-resistant cell lines (Supplementary Figure S8).

## Discussion

Ovarian cancer ranks fifth in cancer deaths among women, accounting for more deaths than any other cancer of the female reproductive system [30]. EOCs have poor prognosis and still remain the most lethal cancers in women. Rationally designed combination therapies provide the best hope of improving outcomes for patients with advanced stage disease [5, 7]. Pharmacological YAP inhibition with VP inhibited tumor cell proliferation and restored sensitivity to CDDP in cells isolated from PDX tumors of urothelial cell carcinoma [31]. Hua et al., [32] proposed a combination therapy with YAP inhibitor (VP) and FGF receptor (BGJ398) to treat fallopian tube and ovarian high grade serous carcinoma. A study by Chen et al., [33] identified that TAZ mRNA and protein are overexpressed in OVCA, and a meta-analysis of an OVCA database indicated that high TAZ mRNA expression correlated with poor prognosis in patients with OVCA. YAP and TAZ are transcriptional coactivators that function as effectors of Hippo signaling pathway. They also identified that TAZ-knockdown resulted in decreased proliferation and migration of OVCA cells, and VP decreased the viability of the OVCA and abolished cell migration. Similar study by Feng et al., [34] concluded that VP treatment of OVCA cells upregulated cytoplasmic YAP and phosphorylation of YAP and downregulated CCN1 and CCN2. These results were corroborated with the significant effect of VP on tumor growth in OVCAR8 xenograft mice, resulting in tumor nodules with lower average weight and reduced volume of gross ascites that they identified. None of the above studies conducted combination treatments of VP with platinum drugs.

Since either CP or CDDP and Taxol are used in combinational chemotherapeutic strategies for OVCA patients, we have chosen to test the efficacy of VP in combination with platinum drugs (CDDP/CP) or Taxol. Both cisplatin-sensitive cells lines (OV-90 and COV504) have shown different IC_50_ values (29.33 μM and 8.38 μM respectively), and surprisingly cisplatin-resistant cell line A2780cis shown the least IC_50_ value of 3.84 μM. Our IC_50_ values are consistent with other published studies of VP. Feng et al., [34] notes IC_50_ 10.55 μM and 17.92 μM in OVCAR3 and OVCAR8 cells respectively. We observed that VP shows synergistic effects with platinum and Taxol in cell lines irrespective of whether they are platinum-sensitive or platinum-resistant. Our studies show that VP when used in combination with CDDP/CP/Taxol shows synergistic activity either in constant or non-constant ratio. The significance of this study is the demonstration of chemo-sensitization of CDDP-resistant cells (A2780Cis) by VP even at lower doses (Fa0.1) of this study. We also observed that downregulation of YAP by VP is associated with decrease in the expression of drug resistance marker ABCG2. Our results agree with previous studies where YAP inhibition with VP inhibited tumor cell proliferation and restored sensitivity to CDDP [31]. Our results also confirm that inhibition of migration and clonogenic capacity is induced by VP treatments. Supporting our previous studies with EMCA [24, 25], VP inhibits cell cycle proteins in OVCA, suggesting the mechanism of inhibition of cell proliferation by cell cycle inhibition. Taken together, we suggest that VP has a role in initial therapy since it improves efficacy of CDDP and that it has a role in drug resistance because it overcomes platinum resistance.

EOC is an immune reactive disease, regulated by various immune cells [35]. Immunologic reaction of EOC plays a significant role in disease control, and hence immunotherapy has emerged as a novel treatment method for EOC [36]. Wang et al., reported that T-helper (Th) cells Th22 and Th17 were significantly increased in EOC patients. There was an increased trend of Th22, IL-22, and TNF-α in stage III–IV patients compared with stage I–II patients [37]. Ovarian cancer ascites is an inflammatory environment that contains a variety of cytokines, chemokines and growth factors [38–40]. Nowak et al. [41] demonstrated that OVCA cells isolated from patients with type II tumors released high levels of immunosuppressive cytokines (i.e., IL-10 and TGF-β). They also observed that cancer cells from patients with type II tumors demonstrated more intense activity in regard to survival and metastasis. Ouh et al., [42] reported that adiponectin treatment of ovarian cancer cells induces angiogenesis via CXC chemokine ligand 1 independently of vascular endothelial growth factor (VEGF) and they suggested that adiponectin may serve as a novel therapeutic target for ovarian cancer. Recently Ni et al., [43] identified that YAP is highly expressed in regulatory T cells (Tregs) and bolsters FOXP3 expression and Treg function in vitro and in vivo. They concluded that YAP could be an amplifier of a Treg-reinforcing pathway with significant potential as an anticancer immunotherapeutic target. In our study, we found elevated levels of cytokines in control OVCA, which suggests a potential role of these cytokines in the development and progression of EOC. These cytokines are efficiently inhibited by VP treatments, suggesting the role of VP in immunotherapeutic development of this drug. Based on our results, we propose that the cytokines of the present study may contribute to the pathology of EOC and may provide novel therapeutic targets.

Chemotherapeutic drug resistance in cancer cells may arise from interactions between cell-intrinsic and tumor microenvironment-mediated mechanisms. The increase in the expression of certain cytokines after VP treatments in CDDP-resistant cells (A2780Cis) is intriguing and questions the validity of VP as a promising immunotherapeutic drug. C-Met is activated by the ligand Hepatocyte Growth Factor (HGF). Activation of the c-Met pathway results in the stimulation of downstream pathways involved in proliferation, scattering, migration, invasion, and survival of tumor cells [44]. The HGF-MET axis is now recognized as playing a vital role in driving VEGF inhibitor resistance [45]. HGF and its physiological receptor tyrosine kinase MET have been reported to be involved in acquired resistance to various tyrosine kinase inhibitors and have been proposed as critical targets in cancer therapy [46]. IP-10 (CXCL10) is an interferon-inducible cytokine that is efficiently induced by IFNβ. CXCL10 and its receptor CXCR3 are increasingly being recognized as pro-tumorigenic in several types of cancers. Elevated serum CXCL10 and increased expression of CXCL10 and CXCR3 in tumor cells have been associated with a poor prognosis and metastasis [47]. Leukemia inhibitory factor (LIF) is a pleiotropic cytokine regulating cell differentiation, proliferation and survival in the embryo and the adult, and is also involved in cancer development. Using quantitative proteomics, Shi et al., [48] systemically investigated paracrine communication between pancreatic stellate cells (PSCs) and pancreatic cancer cells (PCCs) and identified LIF as a critical stromal factor acting on PCCs. Functional studies conducted by them revealed LIF’s physiological significance in driving both tumor progression and chemoresistance. Macrophage Migration Inhibitory Factor (MIF) has been identified as a molecular determinant of the anti-EGFR cetuximab resistance in human colorectal cancer cells [49]. Cisplatin resistant lung cancer cells showed an increased self-renewal ability and promoted M2 polarization of Tumor-associated microphages (TAMs) via the secretion of MIF [50]. TGF-β2 and TGF-β1 can induce CXCR4 expression in several types of tumor cells and leukocytes, via TGF-β type I receptor-dependent non-Smad signaling pathways [51]. They suggest that the intrinsic TGF-β2-triggered SDF-1-CXCR4 signaling axis is crucial for drug resistance dependent on a slow-cycling state in dormant or slow-cycling disseminated tumor cells in bone marrow. TNF-β induces apoptosis and inflammatory signals similar to TNF-α. Studies on ovarian cancer cells demonstrated that TNF-β overexpression is commonly found in different ovarian cancer subtypes, and that the lymphotoxin-β receptor is expressed ubiquitously in ovarian cancer cells as well as cancer-associated fibroblasts. Additionally, in ovarian cancer, TNF-β has been shown to promote tumor-stromal cells interaction in the tumor microenvironment ([52]. Buhrmann et al., [53] demonstrated that resveratrol modulates the TNF-β signaling pathway, induces apoptosis, suppresses NF-κB activation, epithelial-to-mesenchymal-transition (EMT), cancer stem cell-like cells formation and chemosensitizes colorectal cancer cells to 5-Fluorouracil in a tumor microenvironment. Our results corroborate with previous reports, as in our study, in response to VP treatment, A2780Cis cells are inducing the secretion or increasing the expression of above cytokines to increase resistance to VP treatment. Collectively, our results suggest the association of several cytokine signaling pathways that are activated after VP treatment, paving the way to the development of personalized combination therapies for the treatment of chemoresistance in OVCA patients.

Based on the present literature, it is known that the major drawback of current cancer chemotherapy treatments is that the combination of different drugs at higher doses produces drug resistance by cancer cells, undesired toxicity for patients and decreased levels of efficacy. In this study, we suggest that VP can be either synergistic or antagonistic at certain ratios and need to be efficiently administered to obtain optimal therapeutic advantages. Our studies are limited by in vitro model systems and findings that will need to be corroborated in on-going animal and human studies. Further in vivo experiments with different drug combinations and standardization of PK/PD studies in our lab will further contribute to our understanding of therapeutic potential of VP for OVCA patients. Our study provides significant systematic evaluation of VP with CDDP/CP/Taxol interactions for possible application to OVCA patients.

## Conclusions

Our results show that VP is synergistic at certain ratios with either platinum drugs or taxol of the present study. Either in cisplatin-resistant cell lines or cisplatin-sensitive cell lines, VP proves to be more efficient in inhibiting cell proliferation and inducing cytotoxicity. Our results suggest that novel combinations of VP with CDDP or CP or Taxol might be an attractive therapeutic strategy to enhance OVCA chemosensitivity. The fact that lower doses of VP are effective in chemosensitizing the CDDP-resistant cells, might ultimately lead to the development of an innovative combination therapy for the treatment of OVCA patients. Our results suggest that repurposing of VP to be used in combination with cisplatin/carboplatin represents an innovative approach to restore chemosensitivity of ovarian cancer to cisplatin/carboplatin. Further, ongoing in vivo experiments may contribute to our understanding of the mechanism of VP and confirm the therapeutic potential of VP for OVCA patients.

## Supplementary information

**Additional file 1: Figure S1.** Standard curves of drugs in OVCA cells after treatment: MTT assay was done in 96-well plates. In each well 5000 cells were seeded. After 24 h, drug treatments were initiated and given for 72 h and cell proliferation was measured as per Manufacturer’s instructions (Cell Proliferation Kit). DMSO/sterile PBS/sterile water served as controls. Error bars indicate Mean ± SEM. *Statistically significant at *p* < 0.05 (ANOVA), control vs drug treatment. *n* = 9. **Figure S2.** Dose effect curves of drugs in OVCA cells after treatment: Dose effect curves depicting IC_50_ values were constructed following Chou-Talalay method. These were constructed based on MTT assay. **Figure S3.** Standard curves of drugs in EMCA cells after treatment: MTT assay was done in 96-well plates. In each well 5000 cells were seeded. After 24 h, drug treatments were initiated and given for 72 h and cell proliferation was measured as per Manufacturer’s instructions (Cell Proliferation Kit – Sigma). DMSO/sterile PBS/sterile water served as controls. Error bars indicate Mean ± SEM. *Statistically significant at p < 0.05 (ANOVA), control vs drug treatment. n = 9. **Figure S4.** Dose effect curves of drugs in EMCA cells after treatment: Dose effect curves depicting IC_50_ values were constructed following Chou-Talalay method. These were constructed based on MTT assay. **Figure S5.** Combination-index plots of drugs in OVCA cells after treatment: Combination-index plots depicting antagonistic/synergistic drug combinations were constructed following Chou-Talalay method. A – C. Combination index plots in OVCA cell lines. D. Combination index plots in EMCA cell line ARK1. **Figure S6.** Synergistic activity of drugs on COV504 cells in non-constant ratio: IC_50_ values were calculated using Compusyn software following Chou-Talalay method. These calculations were based on MTT assay which was done in 96-well plates. In each well 5000 cells were seeded. The next day, VP and CDDP/CP/Taxol treatments were initiated and given for 72 h and cell proliferation was measured as per Manufacturer’s instructions (Cell Proliferation Kit). DMSO/sterile PBS /sterile water served as control. *n* = 6. VP = Verteporfin; CDDP = cisplatin; CP = carboplatin; Taxol = paclitaxel. After determining cell proliferation (MTT assay) of COV504 cells treated with non-constant ratios of VP and CDDP/CP/Taxol, combination index (CI) values were calculated and represented as heat maps where a drug combination is synergistic (green color) if CI < 1.0; additive (yellow color) if CI = 1.0; and antagonistic (red color) if CI > 1.0. **Figure S7**. Inhibition of clonal formation after drug treatments: Images showing the clones formed after control and drug treatments in OV90 and A2780Cis cells. Experiment is repeated 2 times with at least 3 replicates for each cell line.

**Additional file 2: Figure S8.** OVCA cells were grown and treated with the drugs as described in Methods. Cytokine levels in control and VP-treated samples were determined using human cytokine antibody array as per manufacturer instructions. The membranes were incubated with cell lysates, then processed and assayed using chemiluminescence technique. Data shown are from 5 to 10 s exposures. Spots were analyzed based on the signal intensities using Image studio lite v5.2.

**Additional file 3: Figure S9.** Figure shows full-length blots. Western blots were developed as described in the Methods section. VP = verteporfin; CDDP = cisplatin; CP = carboplatin; PT = paclitaxel.

**Additional file 4: Table S1**. Table showing details of cell lines and reagents used in the study. **Table S2**. Table showing details of drugs used in the study. **Table S3**. Table showing details of Kits and Reagents used in the study. **Table S4A**: Table showing details of primary antibodies used. **Table S4B**: Table showing details of secondary antibodies used. **Table S5**. IC_50_ values (in μM) of EMCA cell lines. **Table S6**. Concentrations (in μM) of the drugs used for the experiments in OVCA cell lines.

## Data Availability

All data generated or analyzed during this study are included in this published article.

## Abbreviations

CDDP: Cisplatin
CI: Combination index
CP: Carboplatin
CTGF: Connective tissue growth factor
DRI: Dose-Reduction Index
EMCA: Endometrial cancer
EOC: Epithelial ovarian cancer
FBS: Fetal bovine serum
HGSOC: High-grade serous ovarian cancer
OS: Overall survival
OVCA: Ovarian cancer
PDT: Photodynamic therapy
PFS: Progression free survival
PT: Paclitaxel (Taxol)
TAZ: Tafazzin
TEAD: TEA Domain Transcription Factor
VP: Verteporfin
YAP: Yes-associated protein
