# Supplementary material for: Synergistic enhancement of efficacy of platinum drugs with verteporfin in ovarian cancer cells
Source: BMC Cancer. 2020 Apr 3;20:273. doi: 10.1186/s12885-020-06752-1 (PMC7318501; doi:10.1186/s12885-020-06752-1)
Supplement: Supplementary file 1 — Additional file 1: Figure S1. Standard curves of drugs in OVCA cells after treatment: MTT assay was done in 96-well plates. In each well 5000 cells were seeded. After 24 h, drug treatments were initiated and given for 72 h and cell proliferation was measured as per Manufacturer’s instructions (Cell Proliferation Kit). DMSO/sterile PBS/sterile water served as controls. Error bars indicate Mean ± SEM. *Statistically significant at p < 0.05 (ANOVA), control vs drug treatment. n = 9. Figure S2. Dose effect curves of drugs in OVCA cells after treatment: Dose effect curves depicting IC50 values were constructed following Chou-Talalay method. These were constructed based on MTT assay. Figure S3. Standard curves of drugs in EMCA cells after treatment: MTT assay was done in 96-well plates. In each well 5000 cells were seeded. After 24 h, drug treatments were initiated and given for 72 h and cell proliferation was measured as per Manufacturer’s instructions (Cell Proliferation Kit – Sigma). DMSO/sterile PBS/sterile water served as controls. Error bars indicate Mean ± SEM. *Statistically significant at p < 0.05 (ANOVA), control vs drug treatment. n = 9. Figure S4. Dose effect curves of drugs in EMCA cells after treatment: Dose effect curves depicting IC50 values were constructed following Chou-Talalay method. These were constructed based on MTT assay. Figure S5. Combination-index plots of drugs in OVCA cells after treatment: Combination-index plots depicting antagonistic/synergistic drug combinations were constructed following Chou-Talalay method. A – C. Combination index plots in OVCA cell lines. D. Combination index plots in EMCA cell line ARK1. Figure S6. Synergistic activity of drugs on COV504 cells in non-constant ratio: IC50 values were calculated using Compusyn software following Chou-Talalay method. These calculations were based on MTT assay which was done in 96-well plates. In each well 5000 cells were seeded. The next day, VP and CDDP/CP/Taxol treatments were initiat [file 12885_2020_6752_MOESM1_ESM.pptx]

## Slide 1
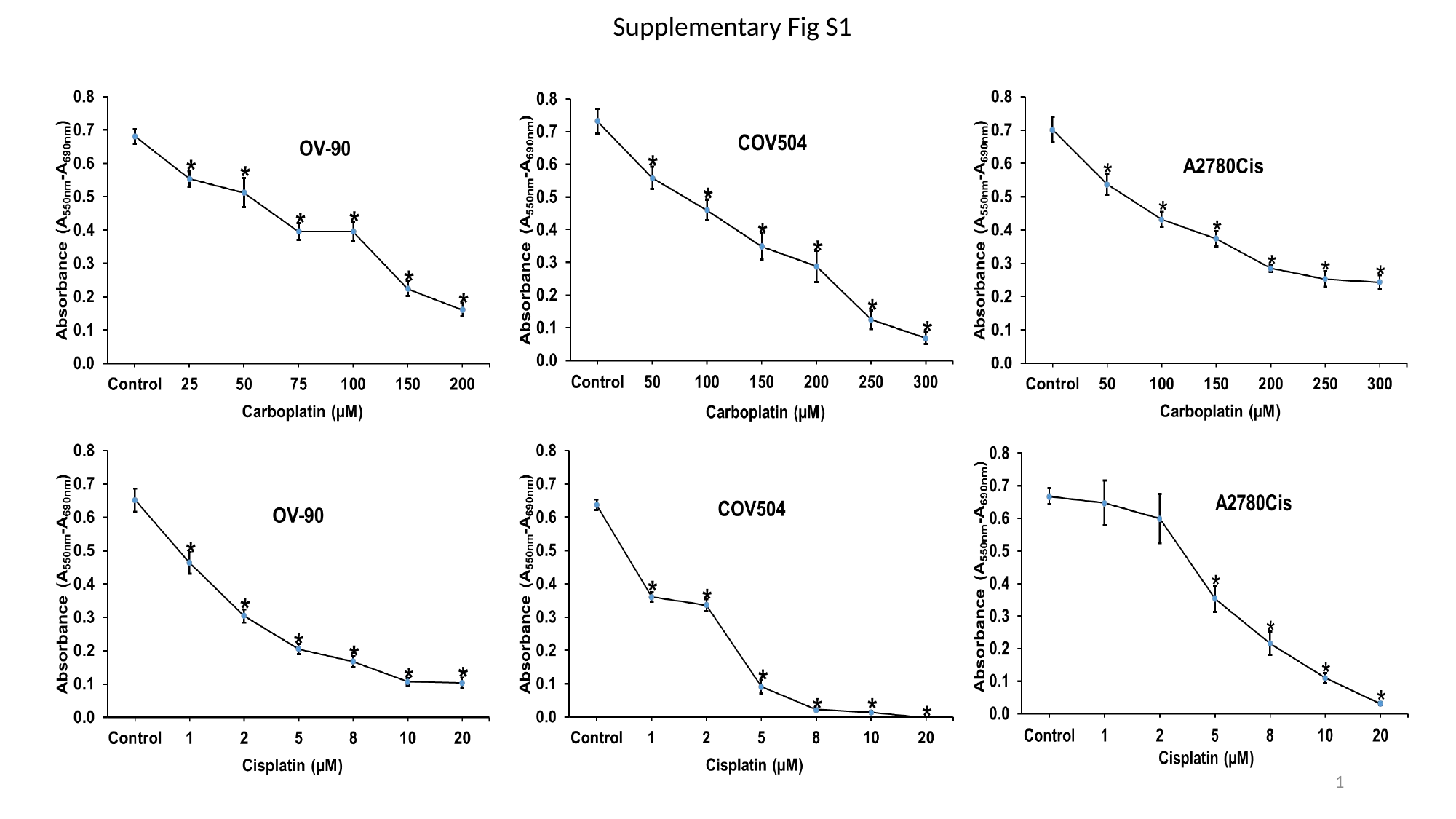

Supplementary Fig S1
1

## Slide 2
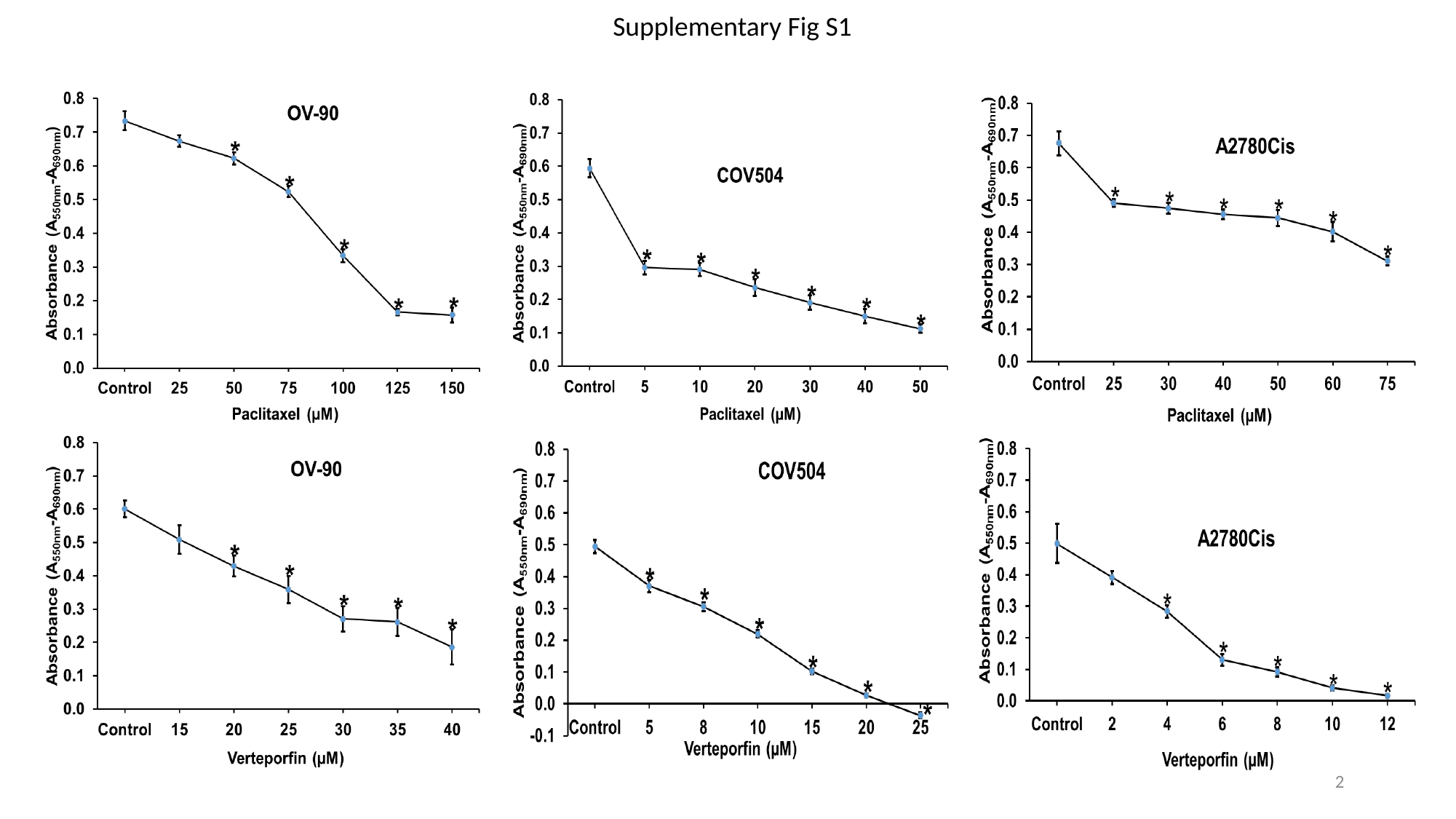

Supplementary Fig S1
2

## Slide 3
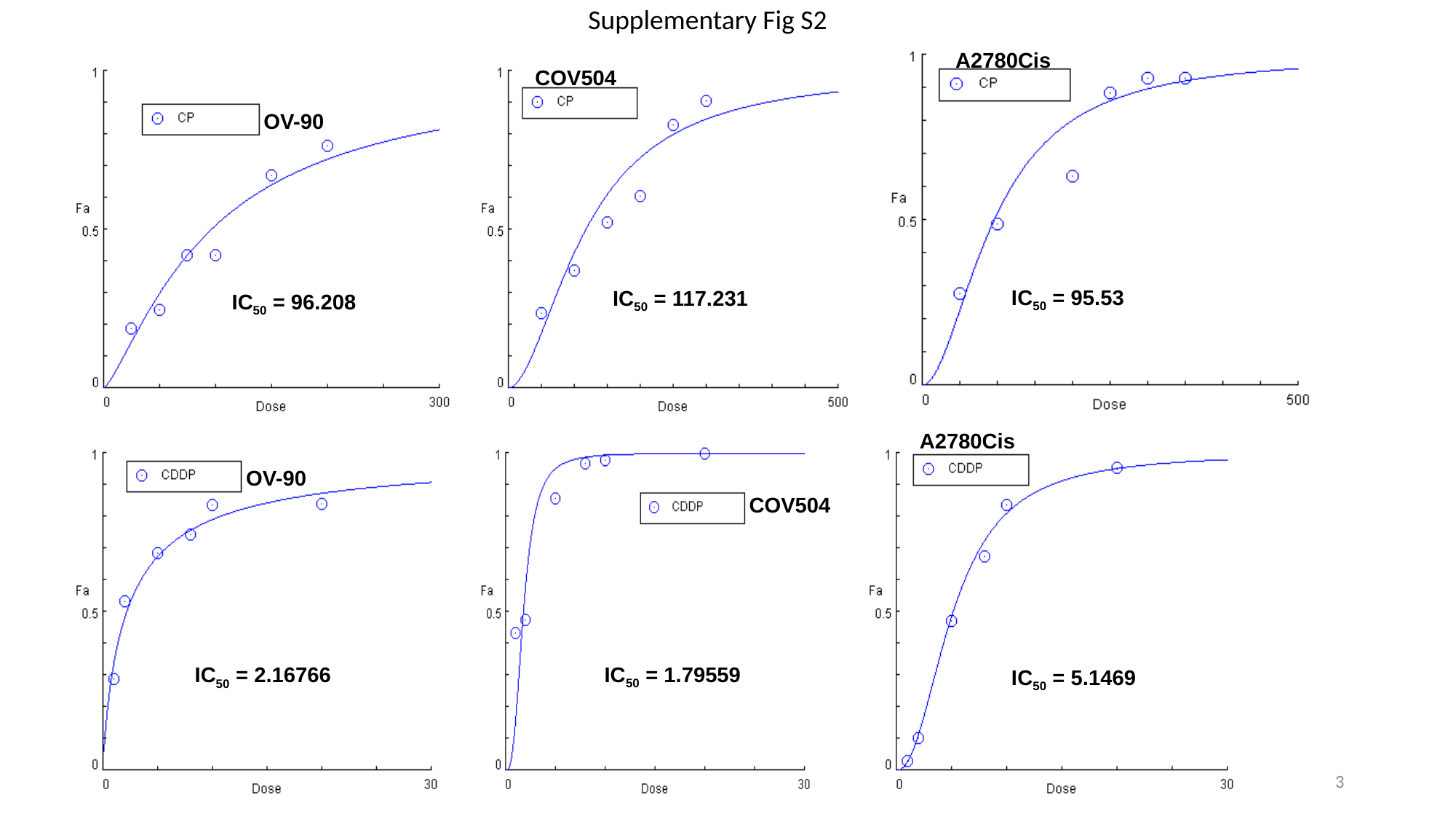

Supplementary Fig S2
IC50 = 96.208
OV-90
A2780Cis
COV504
IC50 = 95.53
IC50 = 117.231
OV-90
IC50 = 2.16766
COV504
IC50 = 1.79559
A2780Cis
IC50 = 5.1469
3

## Slide 4
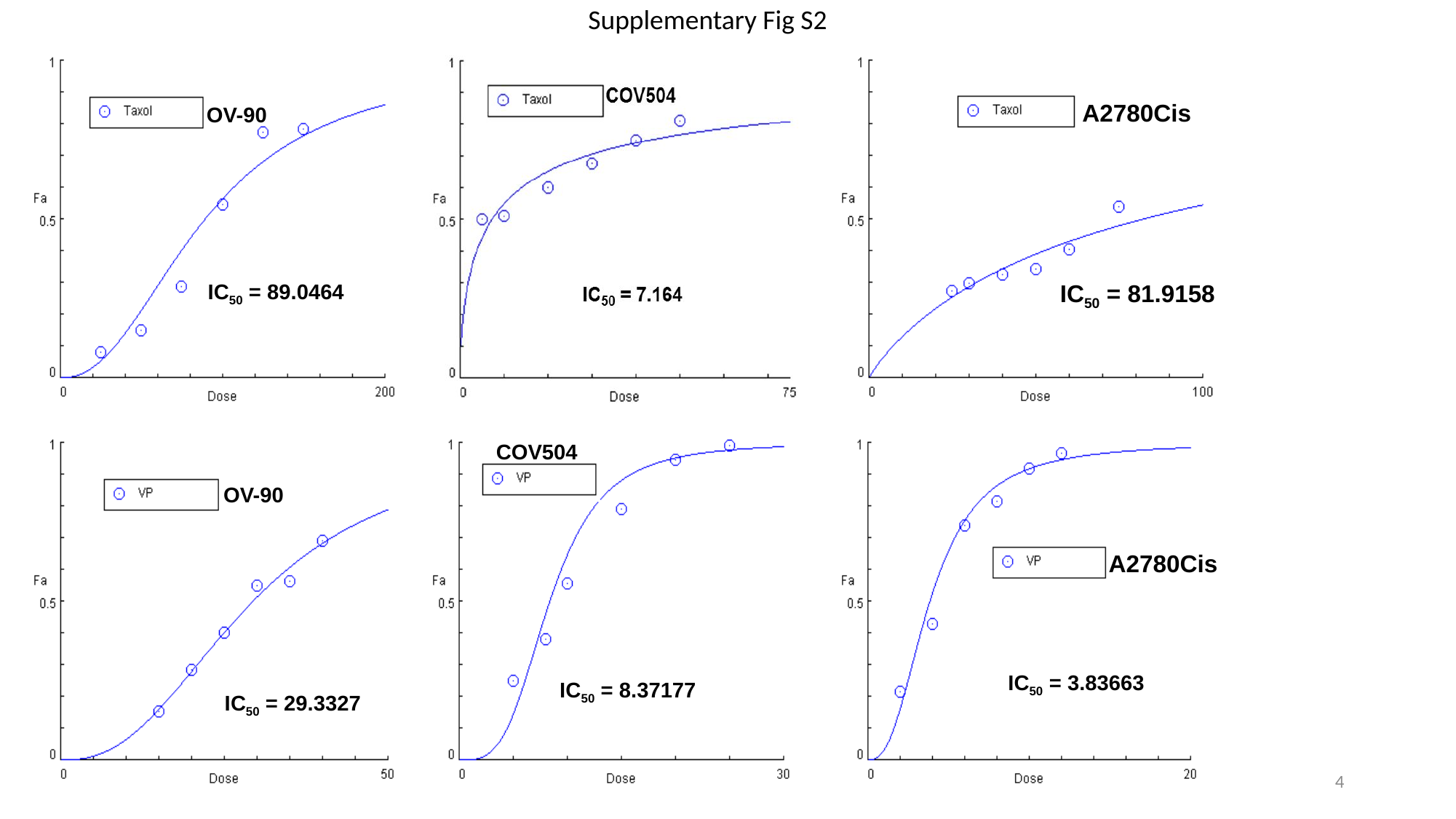

Supplementary Fig S2
OV-90
IC50 = 89.0464
A2780Cis
IC50 = 81.9158
COV504
IC50 = 8.37177
OV-90
A2780Cis
IC50 = 3.83663
IC50 = 29.3327
4

## Slide 5
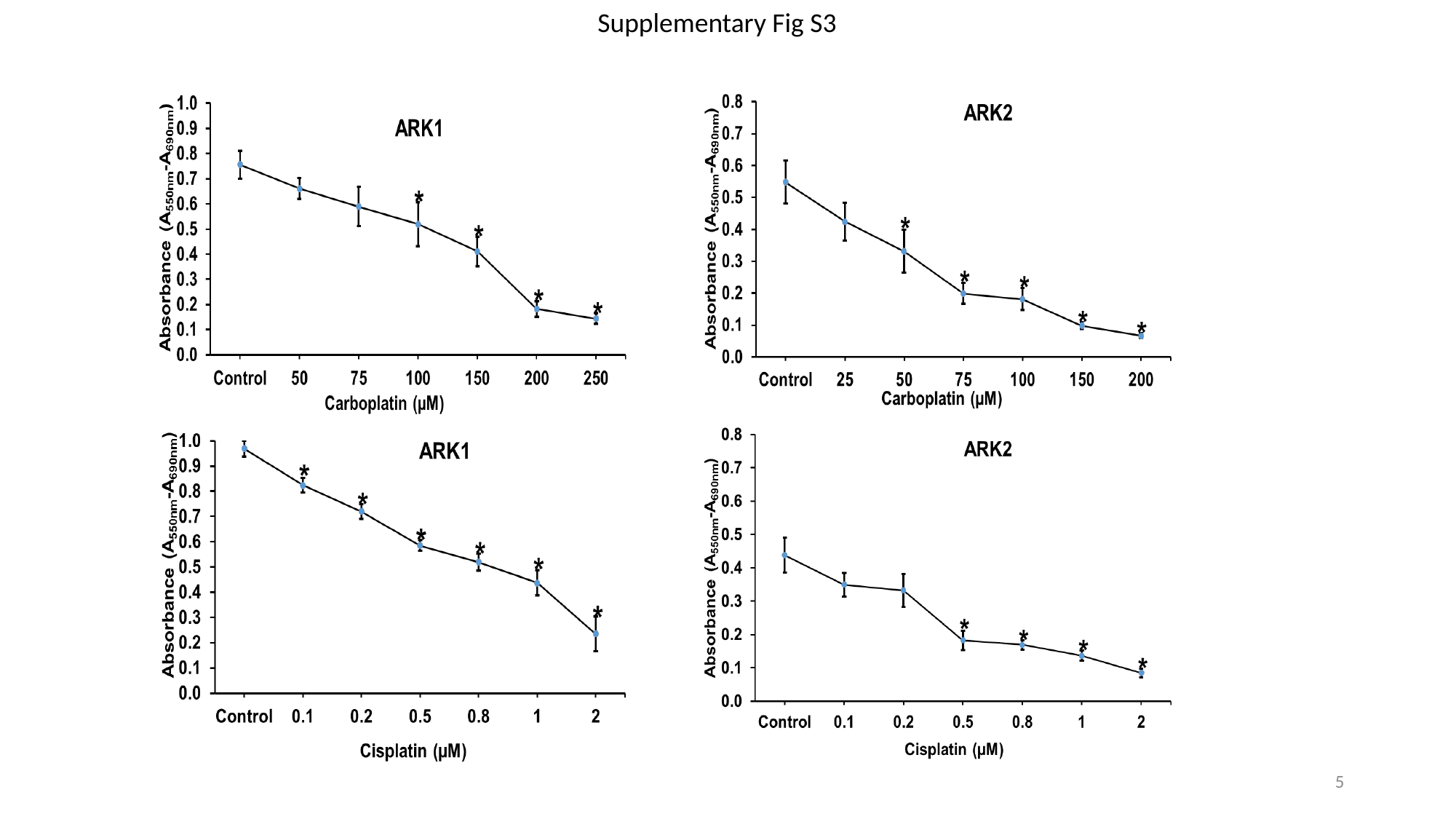

Supplementary Fig S3
5

## Slide 6
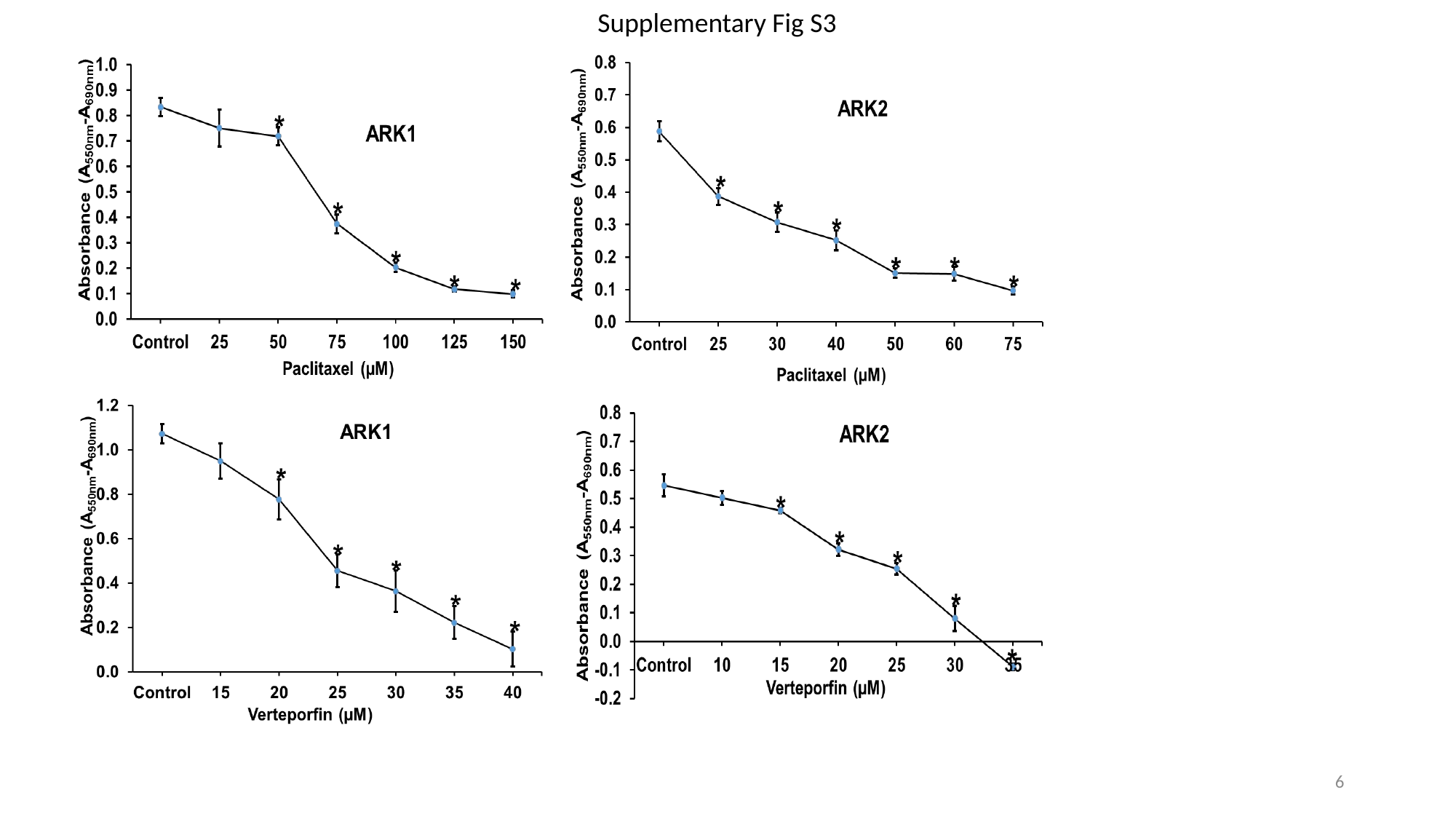

Supplementary Fig S3
6

## Slide 7
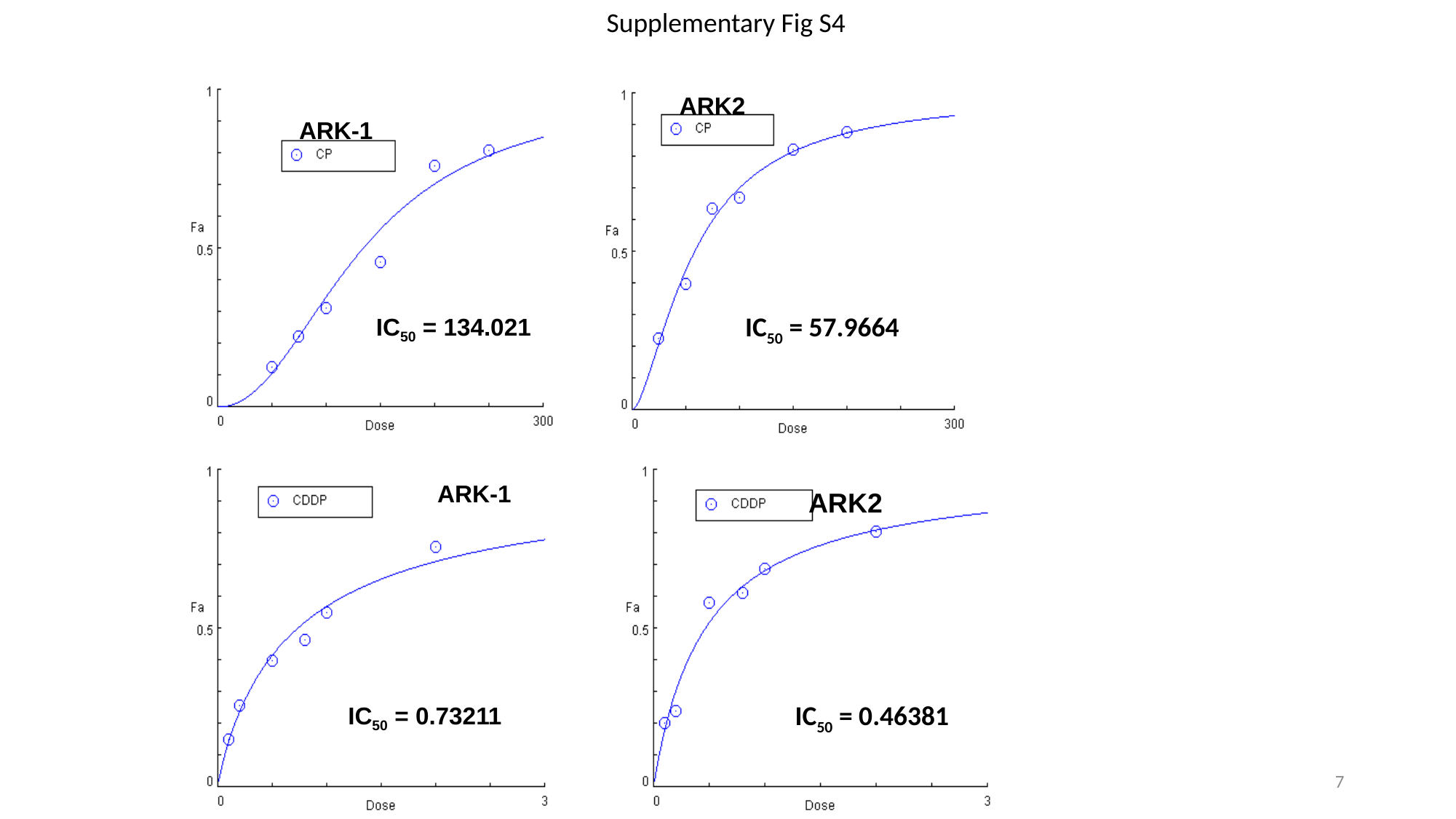

Supplementary Fig S4
ARK-1
IC50 = 134.021
ARK2
IC50 = 57.9664
ARK-1
IC50 = 0.73211
ARK2
IC50 = 0.46381
7

## Slide 8
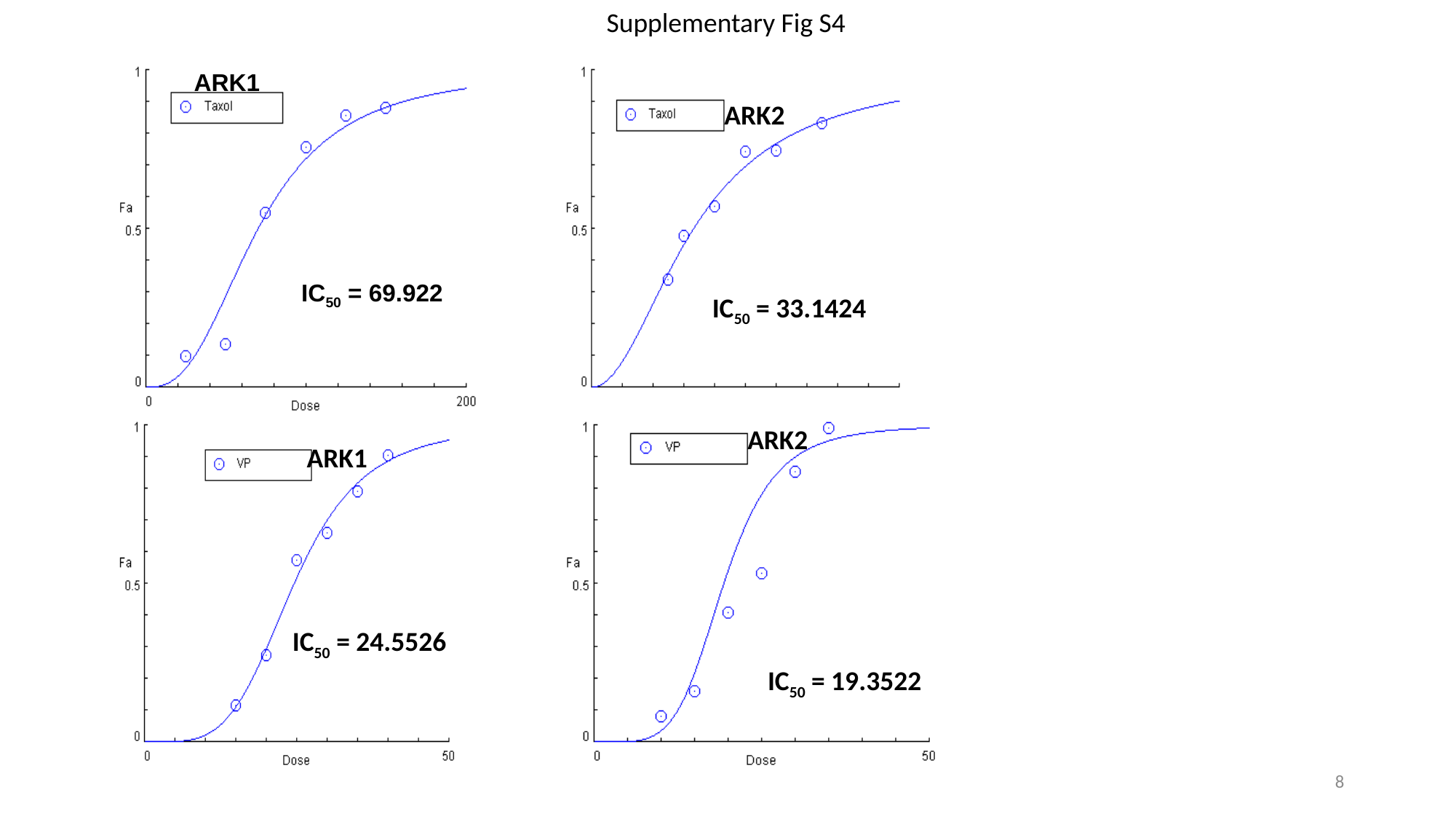

Supplementary Fig S4
ARK1
IC50 = 69.922
ARK2
IC50 = 33.1424
ARK1
IC50 = 24.5526
ARK2
IC50 = 19.3522
8

## Slide 9
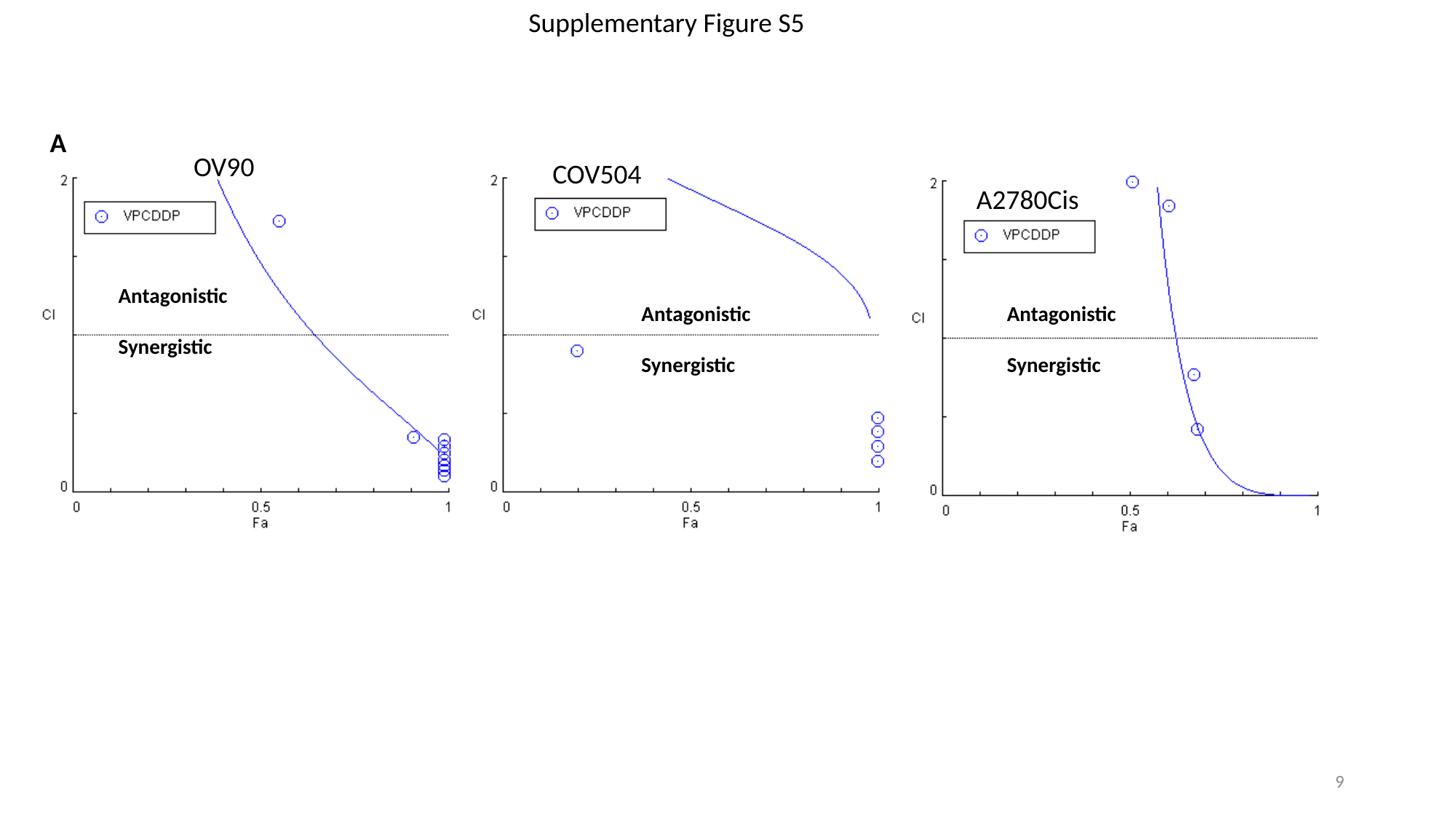

Supplementary Figure S5
A
OV90
COV504
A2780Cis
Antagonistic
Synergistic
Antagonistic
Synergistic
Antagonistic
Synergistic
9

## Slide 10
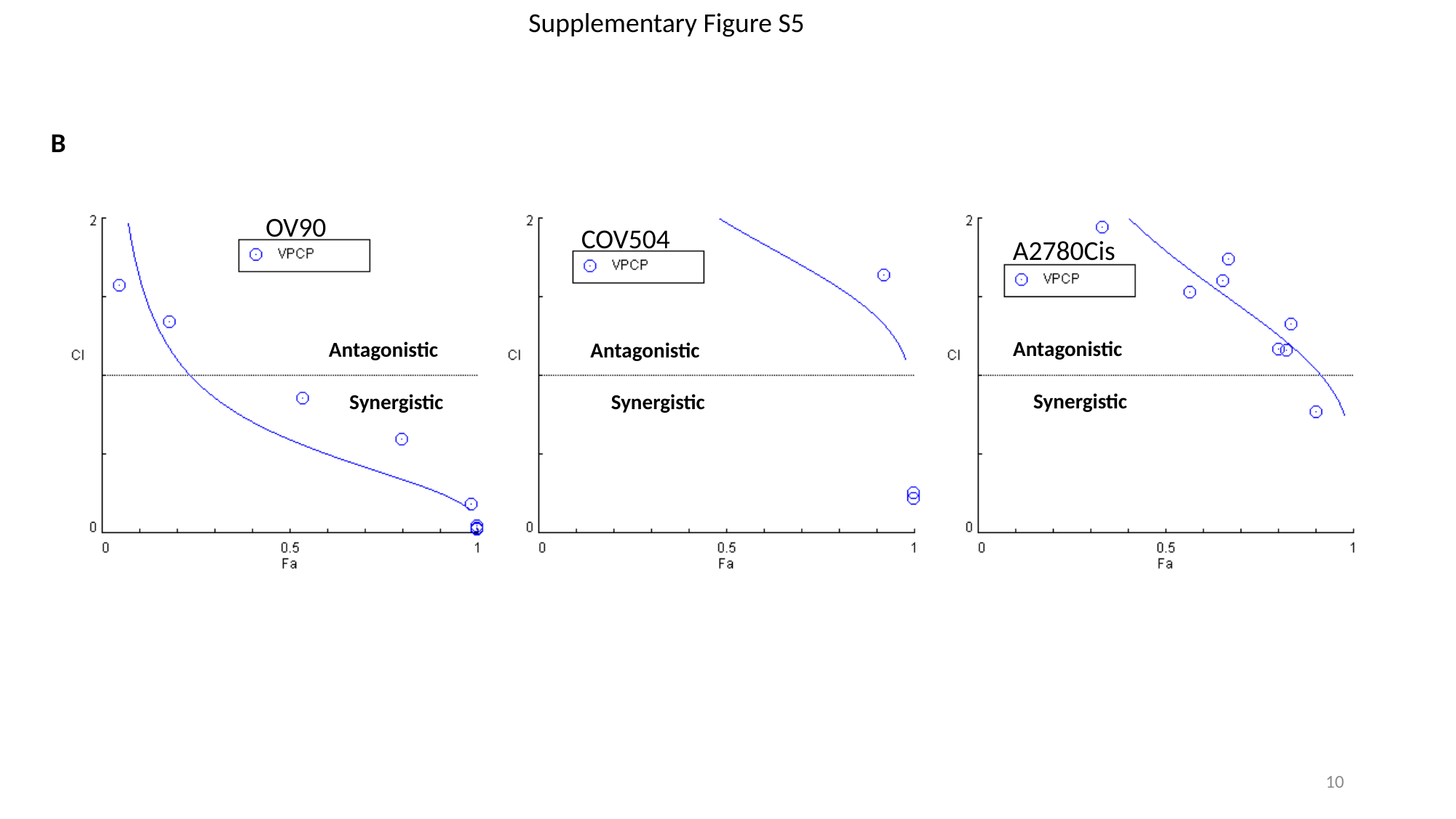

Supplementary Figure S5
B
OV90
COV504
A2780Cis
Antagonistic
Antagonistic
Antagonistic
Synergistic
Synergistic
Synergistic
10

## Slide 11
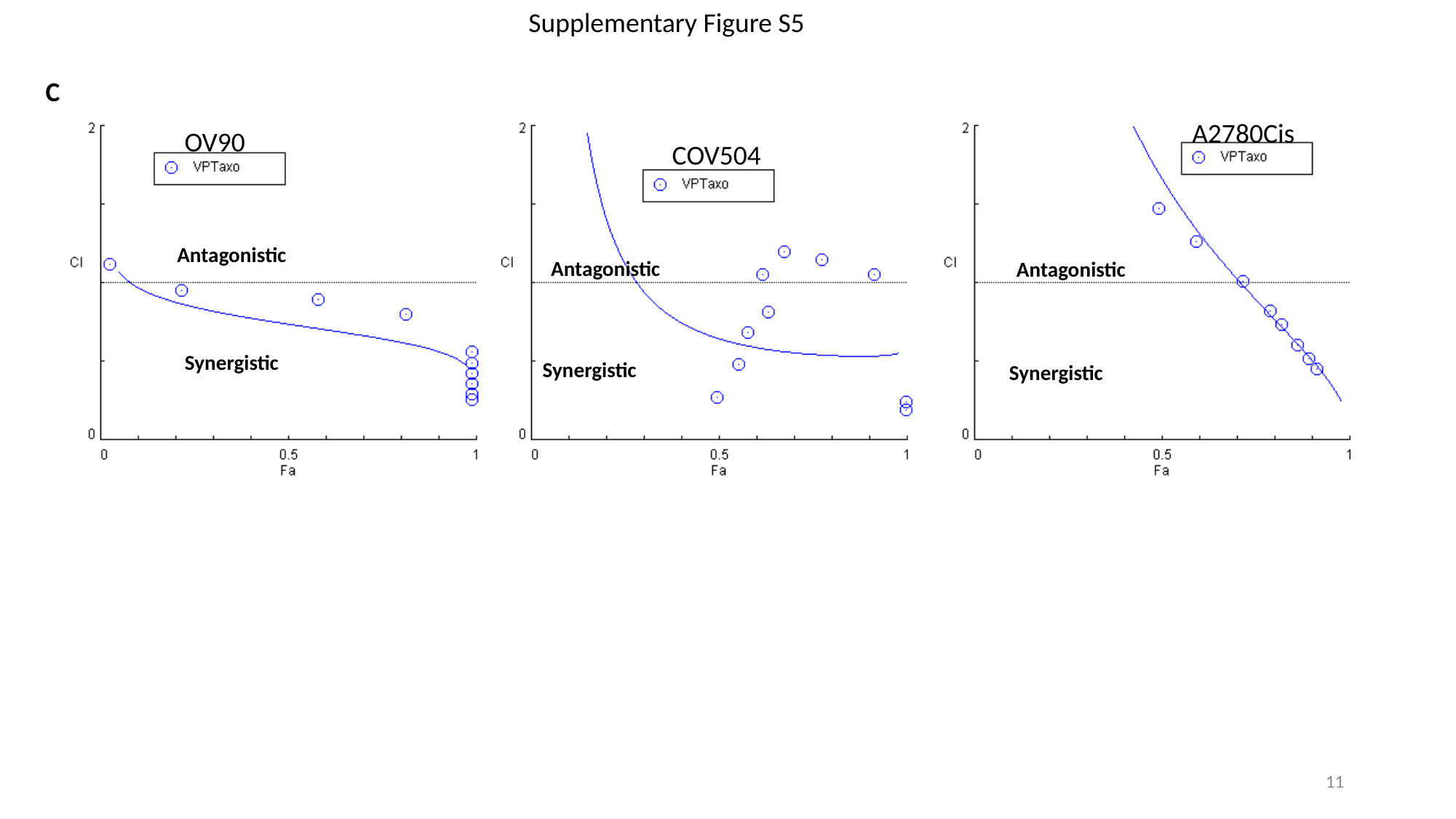

Supplementary Figure S5
C
A2780Cis
OV90
COV504
Antagonistic
Antagonistic
Antagonistic
Synergistic
Synergistic
Synergistic
11

## Slide 12
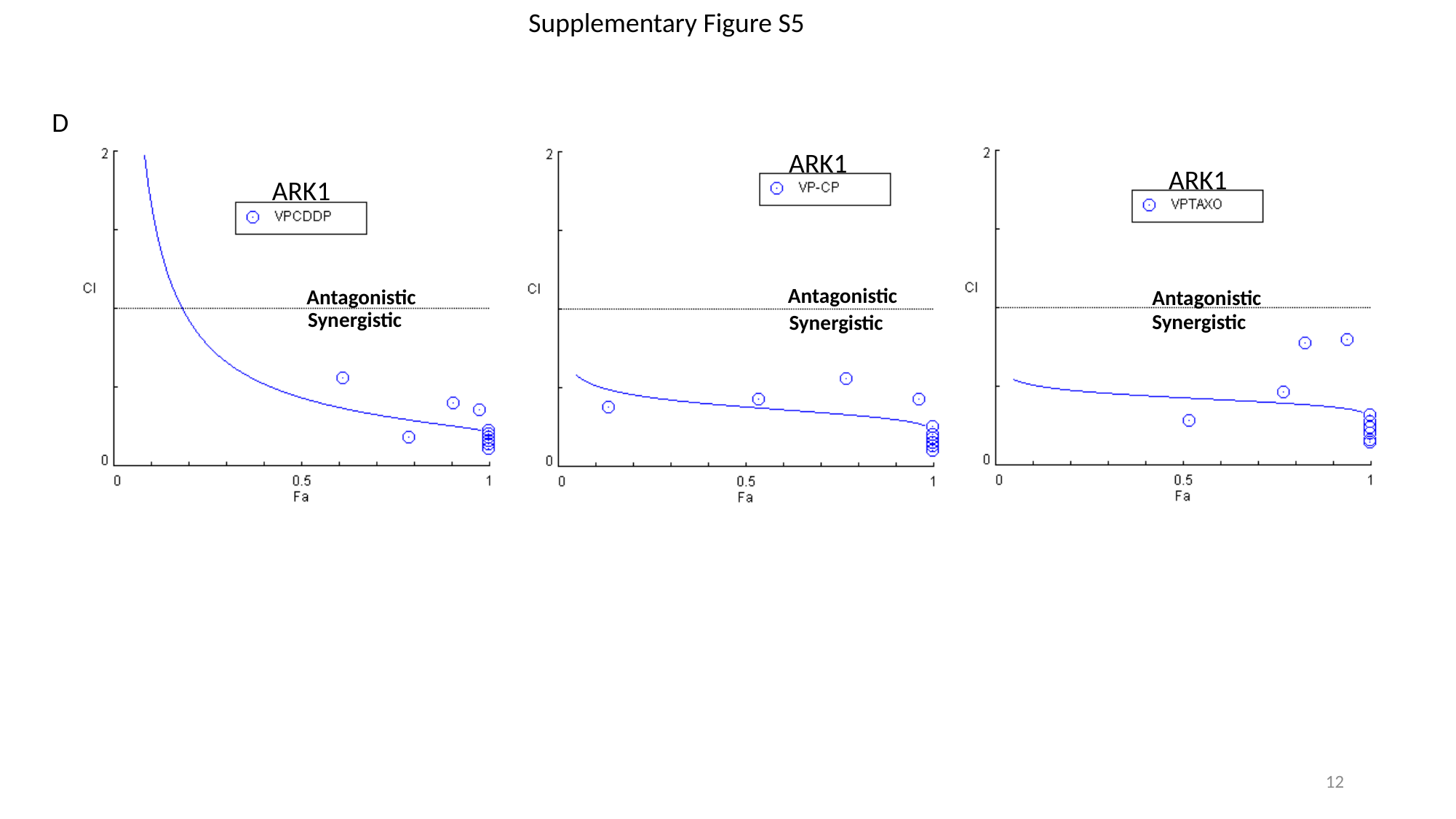

Supplementary Figure S5
D
ARK1
Antagonistic
Synergistic
ARK1
Antagonistic
Synergistic
ARK1
Antagonistic
Synergistic
12

## Slide 13
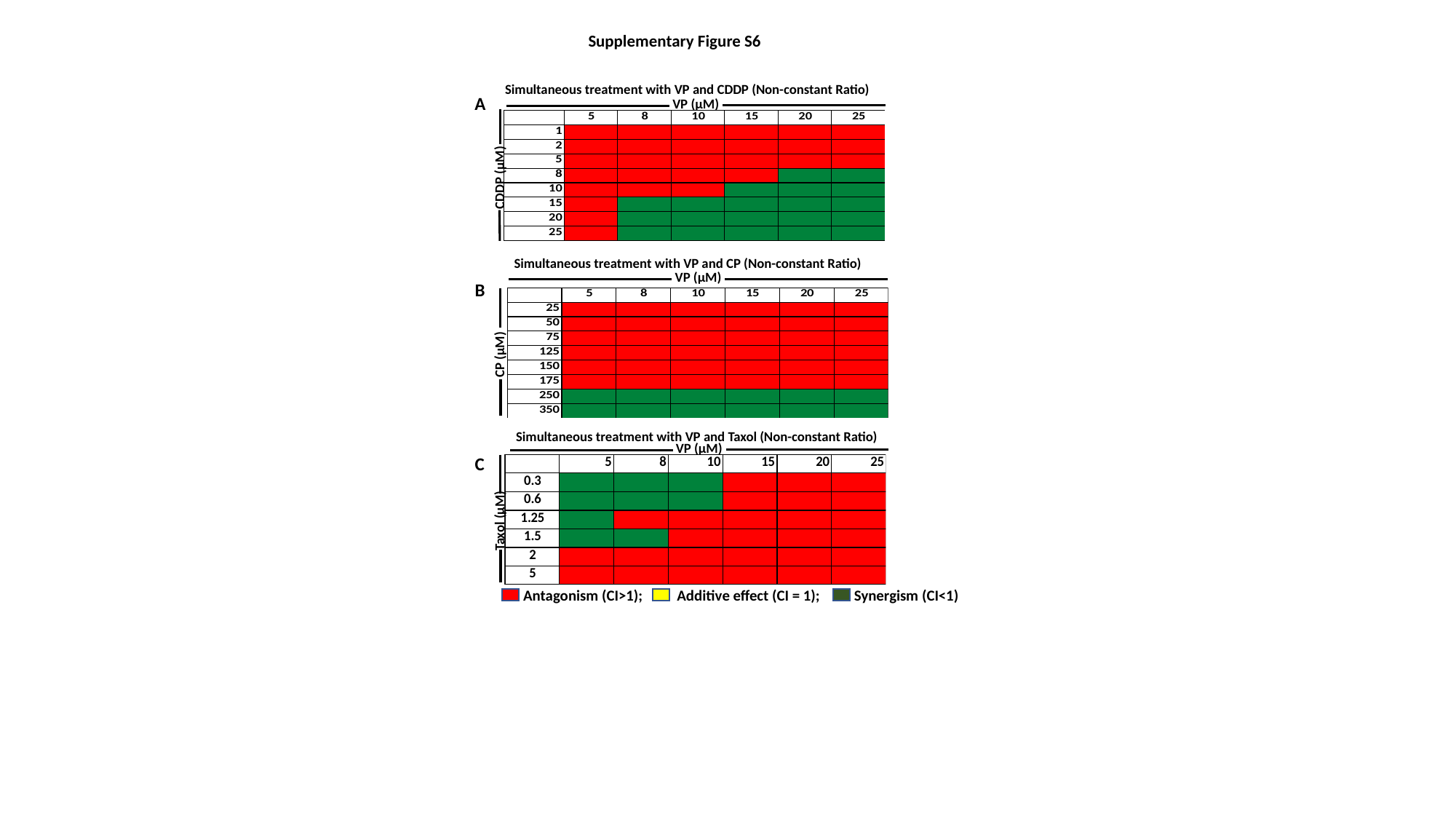

Supplementary Figure S6
Simultaneous treatment with VP and CDDP (Non-constant Ratio)
VP (µM)
CDDP (µM)
A
Simultaneous treatment with VP and CP (Non-constant Ratio)
VP (µM)
CP (µM)
B
Simultaneous treatment with VP and Taxol (Non-constant Ratio)
VP (µM)
Taxol (µM)
C
Antagonism (CI>1); Additive effect (CI = 1); Synergism (CI<1)

## Slide 14
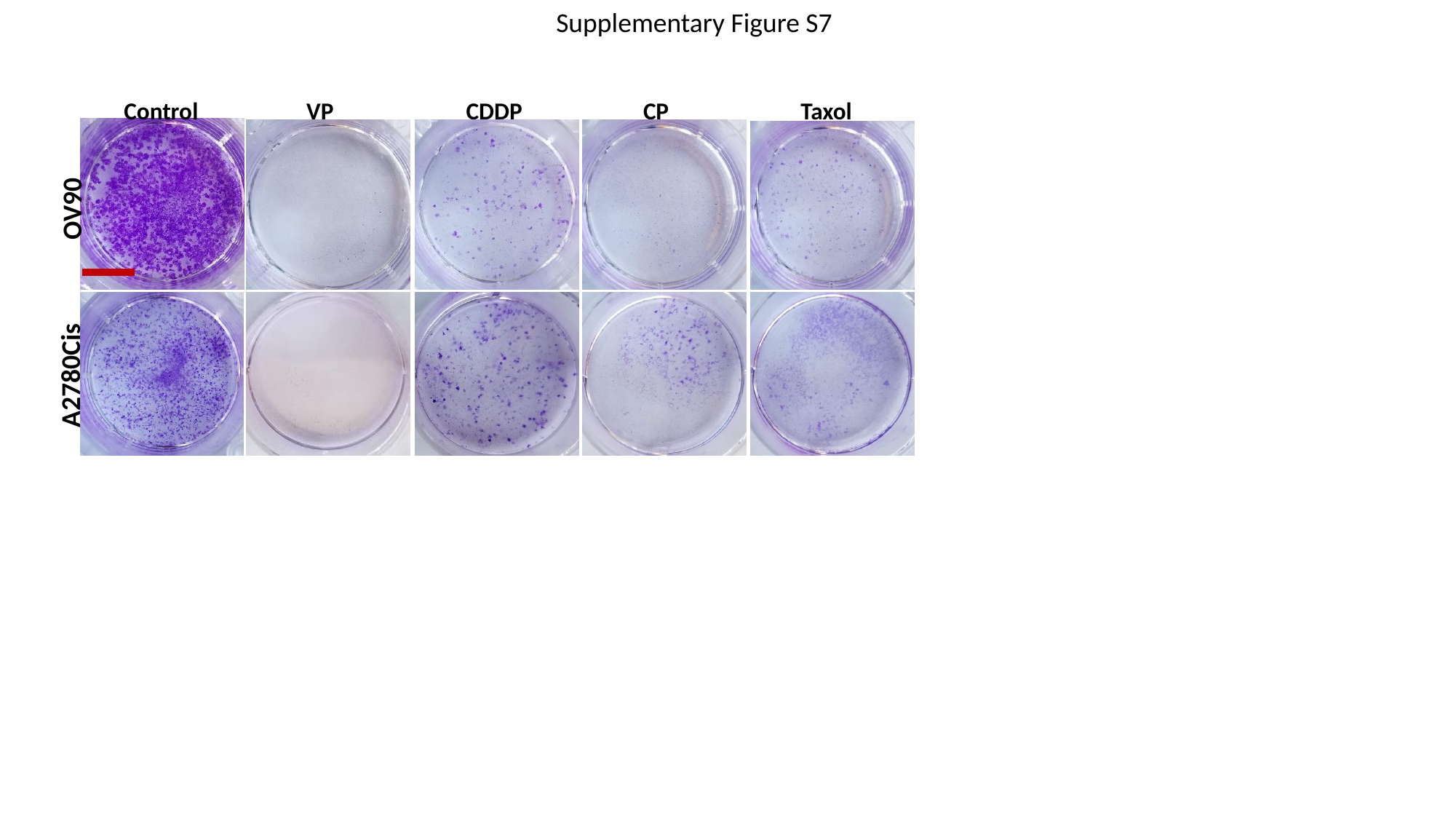

Supplementary Figure S7
Control
VP
CDDP
CP
Taxol
OV90
A2780Cis
