# Supplementary material for: Synergistic enhancement of efficacy of platinum drugs with verteporfin in ovarian cancer cells
Source: BMC Cancer. 2020 Apr 3;20:273. doi: 10.1186/s12885-020-06752-1 (PMC7318501; doi:10.1186/s12885-020-06752-1)
Supplement: Supplementary file 2 — Additional file 2: Figure S8. OVCA cells were grown and treated with the drugs as described in Methods. Cytokine levels in control and VP-treated samples were determined using human cytokine antibody array as per manufacturer instructions. The membranes were incubated with cell lysates, then processed and assayed using chemiluminescence technique. Data shown are from 5 to 10 s exposures. Spots were analyzed based on the signal intensities using Image studio lite v5.2. [file 12885_2020_6752_MOESM2_ESM.pptx]

## Slide 1
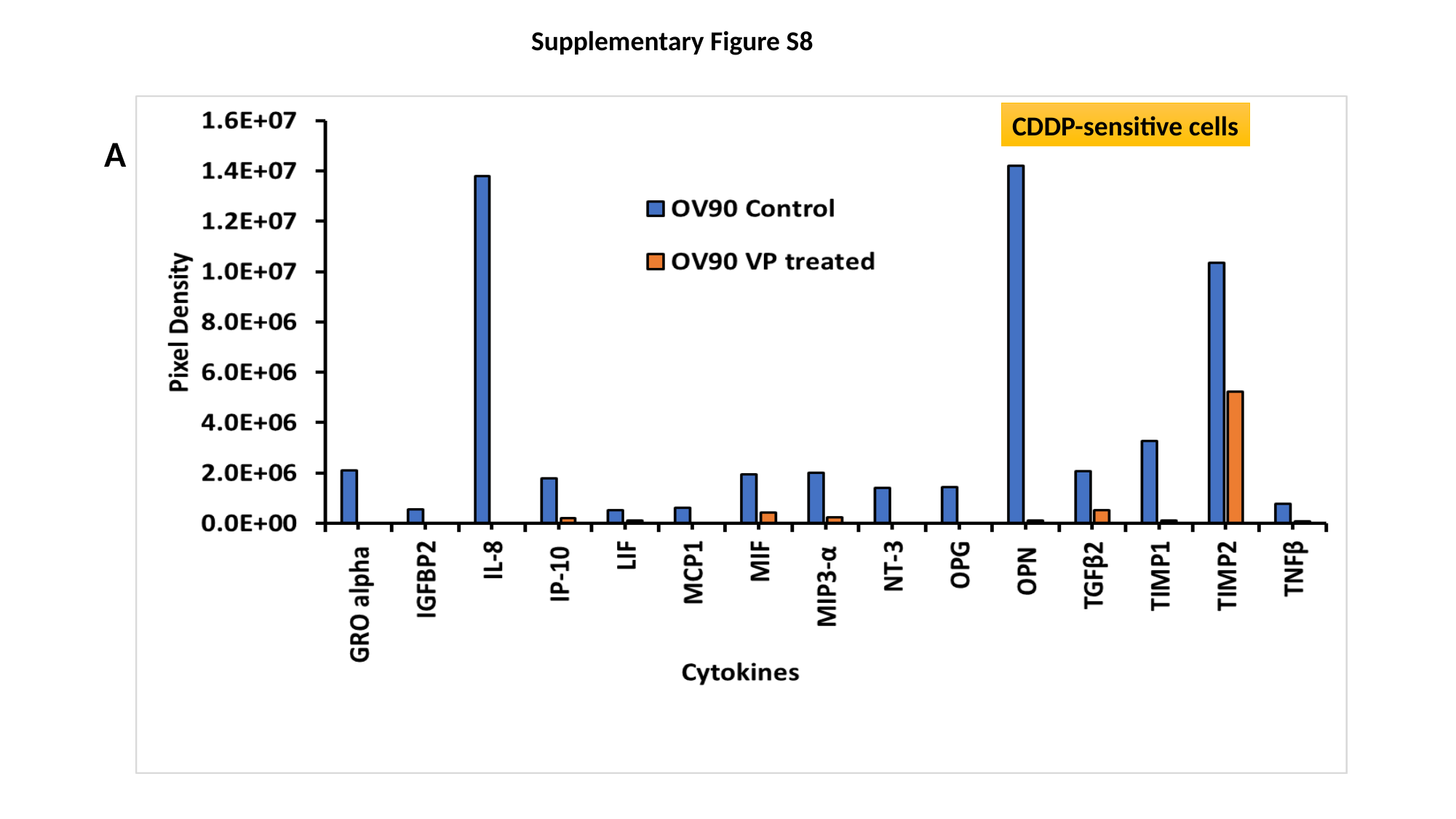

Supplementary Figure S8
CDDP-sensitive cells
A

## Slide 2
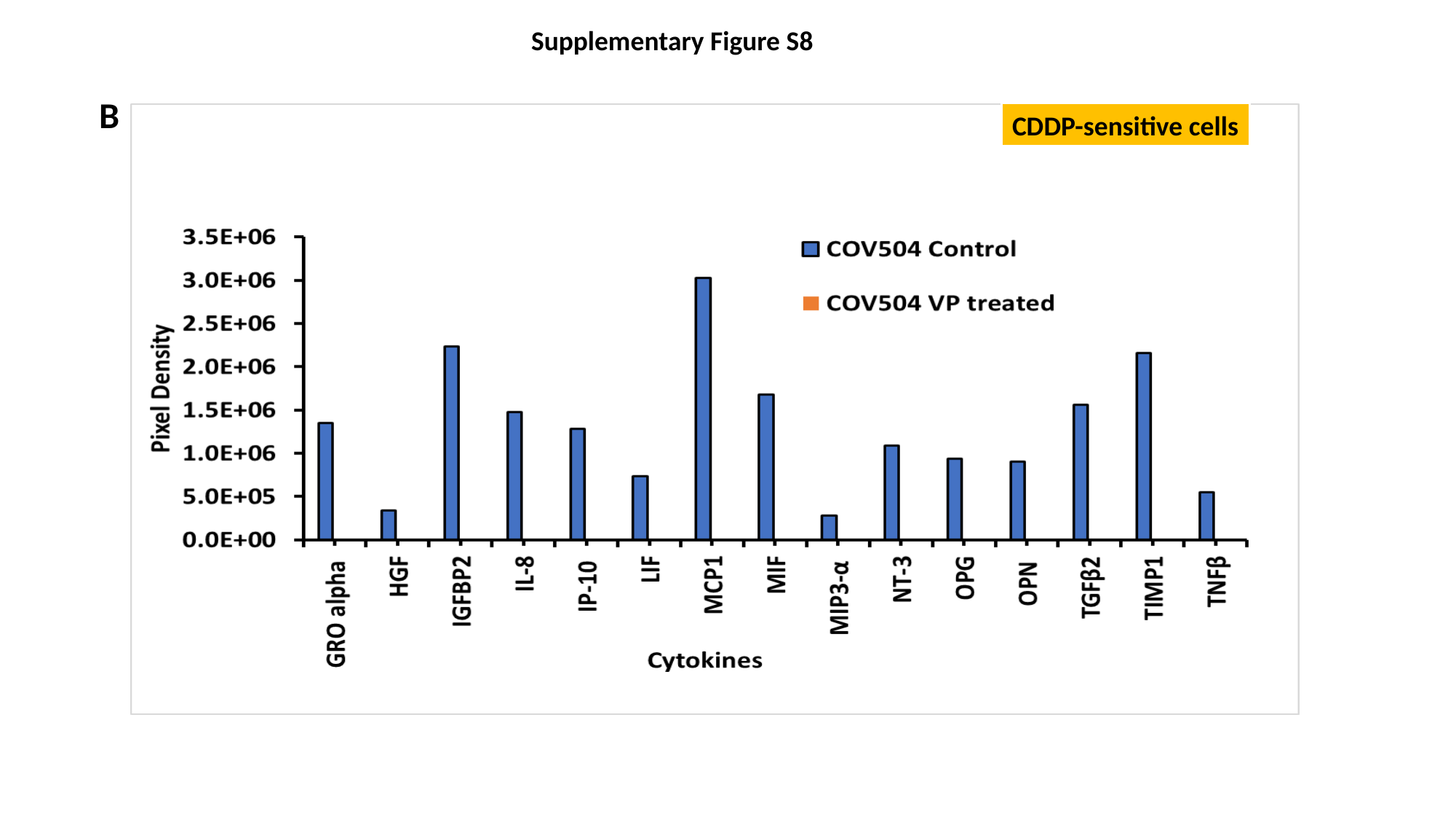

Supplementary Figure S8
B
CDDP-sensitive cells

## Slide 3
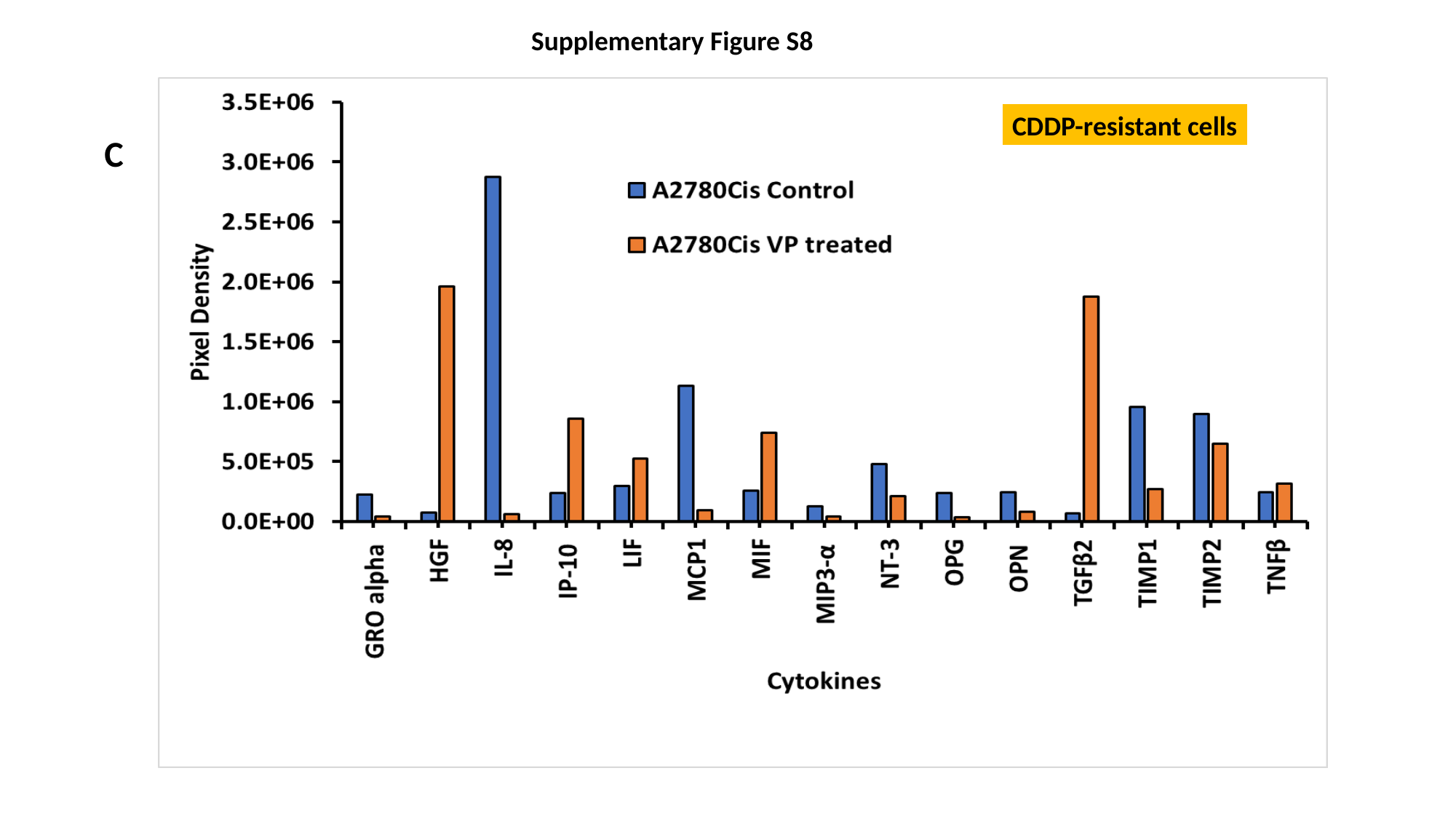

Supplementary Figure S8
CDDP-resistant cells
C
