# Supplementary figures and images for: Synergistic enhancement of efficacy of platinum drugs with verteporfin in ovarian cancer cells
Source: BMC Cancer. 2020 Apr 3;20:273. doi: 10.1186/s12885-020-06752-1 (PMC7318501; doi:10.1186/s12885-020-06752-1)

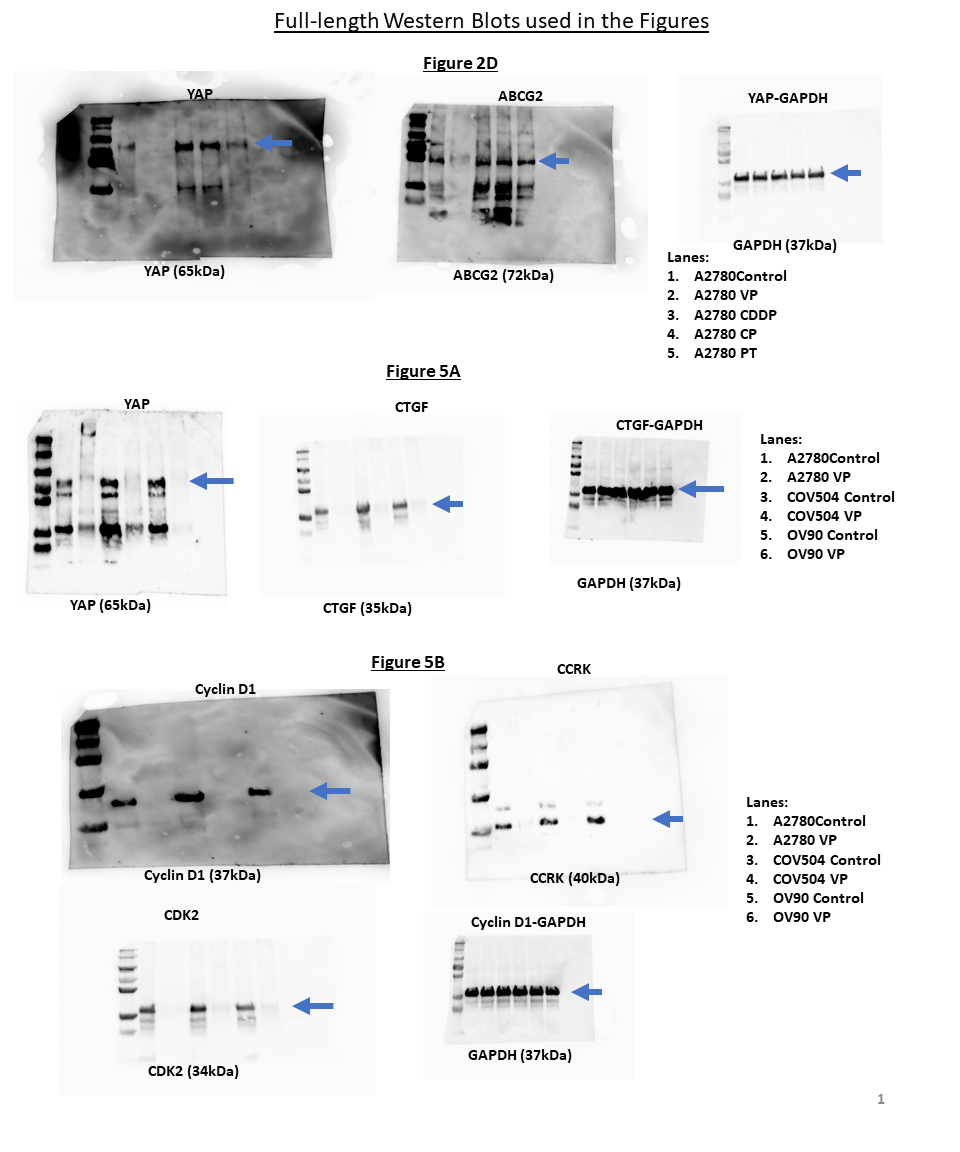

Supplement: Supplementary file 3 — Additional file 3: Figure S9. Figure shows full-length blots. Western blots were developed as described in the Methods section. VP = verteporfin; CDDP = cisplatin; CP = carboplatin; PT = paclitaxel. [file 12885_2020_6752_MOESM3_ESM.tif]
