# Supplementary material for: Synergistic enhancement of efficacy of platinum drugs with verteporfin in ovarian cancer cells
Source: BMC Cancer. 2020 Apr 3;20:273. doi: 10.1186/s12885-020-06752-1 (PMC7318501; doi:10.1186/s12885-020-06752-1)
Supplement: Supplementary file 4 — Additional file 4: Table S1. Table showing details of cell lines and reagents used in the study. Table S2. Table showing details of drugs used in the study. Table S3. Table showing details of Kits and Reagents used in the study. Table S4A: Table showing details of primary antibodies used. Table S4B: Table showing details of secondary antibodies used. Table S5. IC50 values (in μM) of EMCA cell lines. Table S6. Concentrations (in μM) of the drugs used for the experiments in OVCA cell lines. [file 12885_2020_6752_MOESM4_ESM.docx]

Supplementary Table S1. Table showing details of cell lines and reagents used in the study.

**S1a**.

| **Cell lines** | | | | |
| --- | --- | --- | --- | --- |
| Cell line | Obtained from  (date obtained) | Cat. No. | Details | Medium used |
| OV-90 | ATCC  (6/17) | CRL-11732 | Human ovarian cancer cell line, derived from metastatic site from a female patient (Grade 3, Stage IIIc, malignant papillary serous adenocarcinoma). | 1:1 mixture of MCDB 105 medium containing a final concentration of 1.5 g/L sodium bicarbonate and Medium 199 containing a final concentration of 2.2 g/L sodium bicarbonate. |
| COV504 | Sigma  (6/17) | 07071902 | Human ovarian epithelial-serous carcinoma cell line established from a pleural effusion. | DMEM + 2mM Glutamine |
| A2780Cis | Sigma  (3/18) | 93112517 | This cisplatin-resistant cell line has been developed by chronic exposure of the parent cisplatin-sensitive A2780 cell line (Sigma catalogue no. 93112519) to increasing concentrations of cisplatin. The A2780 human ovarian cancer cell line was established from tumor tissue from an untreated patient. | RPMI 1640+ 2 mM Glutamine + 1 µM Cisplatin (add Cisplatin every 2-3 passages). |

Endometrial cancer cells: ARK-1 and ARK-2 were purchased from Dr. Alessandro D. Santin from Department of Obstetrics, Gynecology, and Reproductive Sciences, Yale University School of Medicine, New Haven, CT 06520, USA. Both ARK-1 and ARK-2 are Type 2 Uterine serous papillary carcinoma cells (Ref: PMID: 23359684, PMID: 26333383).

All the cell lines were tested for mycoplasma after every 5 passages.

**S1b.**

| Product description | Obtained from | Cat. No. |
| --- | --- | --- |
| Fetal Bovine serum (FBS) | GE Healthcare | SH30071.03 |
| Antibiotic-Antimycotic | Thermo Fisher Scientific | 15240062 |
| DMEM medium | Thermo Fisher Scientific | 11995073 |
| Medium 199 medium | Thermo Fisher Scientific | 11150059 |
| MCDB 105 medium | Sigma | 117-500 SIGMA |
| RPMI1640, 1X medium | VWR | 16750-070 |

Supplementary Table S2. Table showing details of drugs used in the study.

| **Drug** | **Obtained from** | **Cat. No.** |
| --- | --- | --- |
| Cisplatin | Sigma | 232120 |
| Carboplatin | Sigma | C2538 |
| Paclitaxel | Sigma | T7402 |
| Verteporfin | Sigma | SML0534 |

Supplementary Table S3. Table showing details of Kits and Reagents used in the study.

| **Product** | **Obtained from** | **Cat. No.** |
| --- | --- | --- |
| Nitrocellulose Membrane, Roll, 0.45 µm, 30 cm x 3.5 m | BioRad | 1620115 |
| RIPA Buffer | Boston Bioproducts | BP-115DG |
| Phospho-Protein Extraction Buffer I | Boston Bioproducts | BP-116PB |
| Stripping Buffer 3 | Boston Bioproducts | BP-98 |
| PBS-DULBECCO'S 10X, pH 7.4 | Boston Bioproducts | BB-220DM |
| Tris-Glycine-SDS Running Buffer (10X) | Boston Bioproducts | BP-150 |
| Laemmli (SDS-Sample Buffer, Reducing, 6X) | Boston Bioproducts | BP-111R |
| Laemmli (SDS-Sample Buffer, Non-Reducing, 6X) | Boston Bioproducts | BP-111NR |
| Protease and Phosphatase Inhibitor Cocktail, with EDTA (100X) | Boston Bioproducts | PI-285 |
| CellTiter-Blue® Cell Viability Assay Kit | Promega | G8080 |
| Cell Proliferation Kit I (MTT) | Sigma | 11465007001 |
| Millicell Cell Culture Insert, 12 mm, polycarbonate, 8.0 µm | Sigma | PI8P01250 |
| Lipofectamine™ 3000 Transfection Reagent | Thermo Fisher Scientific | L3000008 |
| PageRuler™ Plus Prestained Protein Ladder, 10 to 250 kDa (2 x 250 µL) | Thermo Fisher Scientific | 26619 |
| TrypLE™ Express Enzyme (1X), phenol red. | Thermo Fisher Scientific | 12605028 |
| Human Cytokine Array C5 | RayBiotech | AAH-CYT-5-8 |

Supplementary Table S4A: Table showing details of primary antibodies used.

| Antigen | Type | Dilution | Manufacturer | Catalogue No. |
| --- | --- | --- | --- | --- |
| ABCG2 | Mouse monoclonal | 1:300 | Santa Cruz | sc-58222 |
| CCRK | Mouse monoclonal | 1:300 | Santa Cruz | sc-517320 |
| CDK2 | Mouse monoclonal | 1:300 | Santa Cruz | sc-6248 |
| CTGF | Rabbit mAb | 1:500 | Cell Signaling Technology | 86641S |
| Cyclin D1 | Mouse monoclonal | 1:300 | Santa Cruz | sc-20044 |
| GAPDH | Mouse monoclonal | 1:1000 | Santa Cruz | sc-365062 |
| YAP | Rabbit polyclonal | 1:500 | Novus Biologicals | NB110-58358 |

Supplementary Table S4B: Table showing details of secondary antibodies used.

| **Type** | **Dilution** | **Manufacturer** | Catalogue No. |
| --- | --- | --- | --- |
| Goat Anti-Rabbit IgG-HRP | 1:5000 | Boston Bioproducts | B-1215 |
| Goat Anti-Mouse IgG-HRP | 1:5000 | Boston Bioproducts | B-1115 |
| m-IgGκ BP-HRP | 1:2000 | Santa Cruz | sc-516102 |

Supplementary Table S5. IC_50_ values (in µM) of EMCA cell lines.

| **Cell lines →**  **Drug↓** | **ARK1** | **ARK2** |
| --- | --- | --- |
| Carboplatin (CP) | 134.021 | 57.9664 |
| Cisplatin (CDDP) | 0.73211 | 0.46381 |
| Paclitaxel (Taxol) | 69.922 | 33.1424 |
| Verteporfin (VP) | 24.5526 | 19.3522 |

Supplementary Table S6. Concentrations (in µM) of the drugs used for the experiments

in OVCA cell lines.

| **Cell lines →**  **Drug↓** | **COV504** | | **OV-90** | | **A2780Cis** | |
| --- | --- | --- | --- | --- | --- | --- |
|  | **Fa0.1** | **Fa0.5** | **Fa0.1** | **Fa0.5** | **Fa0.1** | **Fa0.5** |
| Carboplatin (CP) | 35.19 | 117.23 | 17.74 | 96.21 | 29.89 | 95.53 |
| Cisplatin (CDDP) | 0.84 | 1.80 | 0.17 | 2.17 | 1.88 | 5.15 |
| Paclitaxel (Taxol) | 0.20 | 7.16 | 33.54 | 89.05 | 7.20 | 81.92 |
| Verteporfin (VP) | 4.42 | 8.37 | 12.08 | 29.33 | 1.61 | 3.84 |
